# Distinct Immune Response at 1 Year Post-COVID-19 According to Disease Severity

**DOI:** 10.3389/fimmu.2022.830433

**Published:** 2022-03-21

**Authors:** Chang Kyung Kang, Minji Kim, Jisu Hong, Gwanghun Kim, Soojin Lee, Euijin Chang, Pyoeng Gyun Choe, Nam Joong Kim, Ik Soo Kim, Jun-Young Seo, Daesub Song, Dong-Sup Lee, Hyun Mu Shin, Yong-Woo Kim, Chang-Han Lee, Wan Beom Park, Hang-Rae Kim, Myoung-don Oh

**Affiliations:** ^1^ Department of Internal Medicine, Seoul National University College of Medicine, Seoul, South Korea; ^2^ Department of Biomedical Sciences, Seoul National University College of Medicine, Seoul, South Korea; ^3^ Department of Anatomy & Cell Biology, Seoul National University College of Medicine, Seoul, South Korea; ^4^ BrainKorea21 (BK21) FOUR Biomedical Science Project, Seoul National University College of Medicine, Seoul, South Korea; ^5^ Department of Pharmacology, Seoul National University College of Medicine, Seoul, South Korea; ^6^ Department of Microbiology, School of Medicine, Gachon University, Incheon, South Korea; ^7^ Severance Biomedical Science Institute, Yonsei University College of Medicine, Seoul, South Korea; ^8^ BrainKorea21 (BK21) Project for Medical Science, Yonsei University College of Medicine, Seoul, South Korea; ^9^ College of Pharmacy, Korea University, Sejong, South Korea; ^10^ Medical Research Institute, Seoul National University College of Medicine, Seoul, South Korea; ^11^ Wide River Institute of Immunology, Seoul National University, Hongcheon, South Korea

**Keywords:** SARS-CoV-2, antibody, phagocytosis, memory B cells, memory T cells

## Abstract

**Background:**

Despite the fact of ongoing worldwide vaccination programs for severe acute respiratory syndrome coronavirus 2 (SARS-CoV-2), understanding longevity, breadth, and type of immune response to coronavirus disease-19 (COVID-19) is still important to optimize the vaccination strategy and estimate the risk of reinfection. Therefore, we performed thorough immunological assessments 1 year post-COVID-19 with different severity.

**Methods:**

We analyzed peripheral blood mononuclear cells and plasma samples at 1 year post-COVID-19 in patients who experienced asymptomatic, mild, and severe illness to assess titers of various isotypes of antibodies (Abs) against SARS-CoV-2 antigens, phagocytic capability, and memory B- and T-cell responses.

**Findings:**

A total of 24 patients (7, 9, and 8 asymptomatic, mild, and severe patients, respectively) and eight healthy volunteers were included in this study. We firstly showed that disease severity is correlated with parameters of immune responses at 1 year post-COVID-19 that play an important role in protecting against reinfection with SARS-CoV-2, namely, the phagocytic capacity of Abs and memory B-cell responses.

**Interpretation:**

Various immune responses at 1 year post-COVID-19, particularly the phagocytic capacity and memory B-cell responses, were dependent on the severity of the prior COVID-19. Our data could provide a clue for a tailored vaccination strategy after natural infection according to the severity of COVID-19.

**Graphical Abstract:**
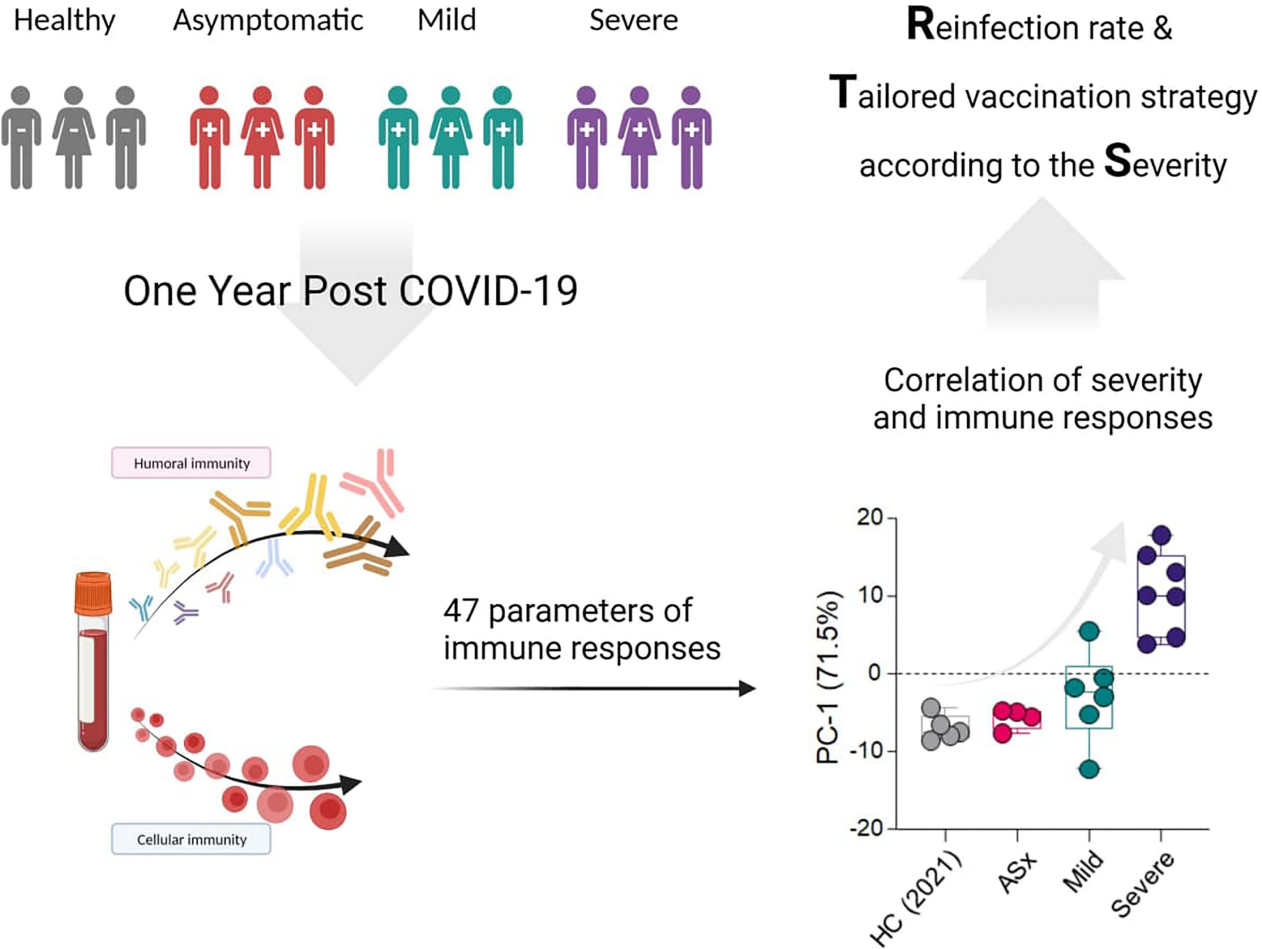


## Introduction

The coronavirus disease 2019 (COVID-19) pandemic is ongoing, with more contagious variants continually emerging (1–3). Despite concerns about reduced effectiveness against new variants, COVID-19 vaccines can substantially reduce both the likelihood of severe acute respiratory syndrome coronavirus 2 (SARS-CoV-2) infection and progression of severe COVID-19 (4–6), making vaccination the most important measure to mitigate the pandemic.

Along with worldwide COVID-19 vaccination programs, following up on immune response after natural SARS-CoV-2 infection is still important to help establish a vaccination strategy for those with a history of SARS-CoV-2 infection (7), predict the protective effect of past infection in those who have not yet received the vaccine (8, 9), estimate and evaluate the longevity of vaccine-induced immune response (10), and explain the milder clinical manifestations of SARS-CoV-2 reinfection relative to the original illness (11).

While reports of reinfection after severe COVID-19 are lacking, there are numerous reports of SARS-CoV-2 reinfection in those who had mild or asymptomatic disease (12–14). Although the exact immunologic mechanism of such phenomenon is uncertain, it must be attributed to the levels of lasting immunity after the original infection. Decreased but persistent humoral and cellular immune responses 8–12 months post-COVID-19 have been reported (15–18). However, thorough integrated immunological assessments especially including Ab-dependent phagocytic capability of plasma from patients for one year post-COVID-19 according to the severity of illness, are scarce.

Therefore, we characterized humoral and cellular immune responses against SARS-CoV-2, namely, levels of Ab isotypes against SARS-CoV-2 antigens, phagocytic capability, and memory B- and T-cell responses 1 year post-COVID-19, according to disease severity. We showed that the breadth and functionality of serologic or memory B and CD4^+^ T cell responses were dependent on the severity of the prior infection, even at 1 year post-COVID-19. These findings suggest that the protective immune response against SARS-CoV-2 can differ according to the severity of COVID-19, which could provide a clue for a tailored vaccination strategy for those who had COVID-19 of varying severity.

## Materials and Methods

### Study Design and Participants

Peripheral blood mononuclear cells (PBMCs) and plasma samples of patients with laboratory-confirmed COVID-19 (by reverse-transcription polymerase chain reaction) had been collected (19). We analyzed PBMCs 12 months after diagnosis (asymptomatic patients) or disease onset, and plasma samples at 8 and 12 months post-COVID-19.

Clinical characteristics, namely, age, sex, and day of onset or diagnosis of COVID-19, and also details of oxygen and medical therapy, were obtained from the electronic medical records. Asymptomatic patients were those with a body temperature <37.5°C and absence of symptoms during their stay in the community treatment center, with a systematic medical interview twice a day (20). All of patients were diagnosed *via* contact tracing during the Daegu metropolitan city outbreak in March 2020 (21). Symptomatic cases were classified as severe when a patient had radiological pneumonia and decreased oxygen saturation (≤93%) in ambient air during their illness; otherwise, they were classified as mild cases (22).

We also analyzed the PBMCs and plasma of SARS-CoV-2 seronegative, unvaccinated healthy control during the pandemic period (HC [2021]) group who had neither received a COVID-19 exposure notification nor been diagnosed with COVID-19 (23).

The Institutional Review Board of Seoul National University Hospital approved the study (IRB No. H-2004-158-1118), and written informed consent was obtained from all participants, in accordance with the Declaration of Helsinki.

### Cells and Antibodies

THP-1 cells were purchased from the American Type Culture Collection (ATCC; Manassas, VA, USA) and maintained in RPMI 1640 media (WELGENE, Gyeongsan-si, Republic of Korea) containing 2 mM L-glutamine, 10% fetal bovine serum (FBS; Thermo Fisher Scientific, Waltham, MA, USA), 50 μM β-mercaptoethanol (Merck Millipore, Billerica, MA, USA), and 1× antibiotic-antimycotic solution (Thermo Fisher Scientific). Cell culture densities were kept below 1 × 10^6^ cells/ml to maintain consistent assay performance.

Murine anti-human IgM (Sigma-Aldrich, Saint Louis, MO, USA), IgA (PROGEN Biotechnik GmbH, Heidelberg, Germany), IgG_1_ (Abcam, Cambridge, UK), IgG_2_ (BioLegend, San Diego, CA, USA), IgG_3_ (BioLegend), and IgG_4_ (BioLegend) Abs, anti-mouse IgG (H + L)-conjugated with horseradish peroxidase (HRP; Thermo Fisher Scientific), and anti-human IgG (Fc)-conjugated with HRP (Arigobio, Hsinchu, Taiwan) were purchased.

### Preparation of Recombinant SARS-CoV-2 Antigens

Genes encoding receptor-binding domain (RBD)-6×his were cloned in-frame into the mammalian expression vector pcDNA3.4 using Gibson Assembly cloning (NEB, Ipswich, MA, USA). SARS-CoV-2 spike protein RBD-monomeric Fc (mFc) and angiotensin-converting enzyme 2 (ACE2)-mFc expressing plasmids were obtained from Prof. J. McLellan (University of Texas, Austin, TX, USA) (24). SARS-CoV-2 antigens and ACE2-mFc were produced in Expi293 cells (Thermo Fisher Scientific), as described previously (25). mFc-tagged proteins were purified by Protein A high-capacity agarose resin (Thermo Fisher Scientific) affinity chromatography. Then, 25× phosphate-buffered saline (PBS) was added to filtered supernatants to a 1× concentration, and the mixture was passed over the column three times. The column was washed with 100 ml of 1× PBS to remove nonspecific bound proteins. Then, 3 ml of 100 mM glycine-hydrochloride (pH 2.7) was added to elute the bound proteins, and the elution was immediately neutralized with 1 ml of 1 M Tris–Cl (pH 8.0). Samples were buffer-exchanged into pH 7.4 PBS using Amicon Ultra-4 (Merck Millipore, Burlington, MA, USA) spin columns with a 10 kDa cutoff. The purity of purified samples was assessed by 12% SDS-PAGE gel. His–tagged RBD was purified according to the instruction of the manufacturer using Ni-NTA agarose resin (Thermo Fisher Scientific) affinity chromatography (26).

Other SARS-CoV-2 antigens, namely, spike (Sino Biological, Wayne, PA, USA), nucleocapsid (NC; Prosci, Fort Collins, CO, USA), and membrane (M) protein (MRC PPU, Dundee, UK), were purchased.

### ELISA

For each SARS-CoV-2 antigen, 100 ng was coated on a 96-well polystyrene ELISA plate (Thermo Fisher Scientific) overnight at 4°C. After blocking with 1× PBS (pH 7.4) containing 3% bovine serum albumin (BSA) for 1 h at room temperature, the plate was incubated with diluted plasma (1:100) at room temperature for 1 h. After washing four times with the PBST buffer (PBS with 0.05% Tween-20), either diluted mouse anti-human immunoglobulin M (IgM, 1:5,000), IgA (1:100), IgG_1_ (1:1,000), IgG_2_ (1:200), IgG_3_ (1:200), or IgG_4_ (1:200) Abs were added and incubated for 1 h. After washing four times with PBST, anti-mouse IgG (H + L)-conjugated with HRP (Thermo Fisher Scientific) was added and incubated for 1 h. For the detection of total IgG, mouse anti-human IgG Fc-conjugated with HRP (Arigobio) was added and incubated for 1 h. After washing four times with the PBST buffer, 50 μl of 3,3′,5,5′-tetramethylbenzidine substrate was added per well (Thermo Fisher Scientific); 50 μl of 2 M H_2_SO_4_ was added to neutralize, and the absorbance at 450 nm was measured using the Infinite 200 PRO NanoQuant microplate readers (Tecan Trading AG, Männedorf, Switzerland).

### Phagocytosis Assays

Protein antigens were covalently coupled to fluorescent beads *via* a two-step carbodiimide reaction. The beads were activated with 40 μl of activation buffer (0.1 M NaH_2_PO_4_, pH 6.2), 5 μl of 50 mg/ml Sulfo-NHS(N-hydroxysulfosuccinimide) (A39269; Pierce, Appleton, WI, USA), and 3.35 μl of 75 mg/ml 1-ethyl-3-(3-dimethylaminopropyl)carbodiimide (EDC) and incubated for 30 min at room temperature. The beads were washed three times in coupling buffer (100 mM sodium citrate, pH 4.0) and then incubated with protein antigen in coupling buffer for 2 h at room temperature. The beads were subsequently washed and blocked with PBSA (PBS with 0.1% BSA, pH 7.4), and then washed with PBS-TBN (PBS with 0.1% BSA, 0.05% sodium azide, 0.02% Tween-20, pH 7.4). The beads were resuspended in 1 ml of 5% BSA/PBS, incubated overnight at 4°C, and then washed and resuspended in 1 ml of PBS.

In order to assess the phagocytic capability of plasma from patient, we conducted *in vitro* phagocytosis assays with human monocyte cell line (THP-1) cells. The antigen-coated beads were incubated with plasma for 20 min and added to 96-well plates so that each well contained 1 × 10^6^ beads per well. In addition, THP-1 cells were seeded into 96-well plates at 1 × 10^5^ cells/well and incubated for 2 h at 37°C with 5% CO_2_. Phagocytosis was evaluated by a BD FACSCanto™ system (BD Biosciences, San Jose, CA, USA) and reported as the fraction of bead-positive cells relative to the total number of THP-1 cells in the sample. Data were analyzed using FlowJo software (version 10.7.1; TreeStar Inc., Ashland, OR, USA).

### Peripheral Blood Sample Processing and Culture

PBMCs were purified from heparinized peripheral whole blood using a Ficoll–Histopaque gradient (1.077 g/ml; GE Healthcare Life Sciences, Piscataway, NJ, USA). They were stored in liquid nitrogen in a freezing medium consisting of 50% FBS, 10% dimethyl sulfoxide (DMSO), and 40% RPMI-1640 (all from Thermo Fisher Scientific) until analysis (27). Cells were cultured in complete RPMI-1640 containing 10% FBS and 1× penicillin/streptomycin (Thermo Fisher Scientific) and stimulated as follows.

### Detection of SARS-CoV-2-Specific T Cells Using Activation-Induced Markers

Antigen-specific memory T cells were measured as a percentage of activation-induced markers (AIM)-expressing CD4^+^ T and CD8^+^ T cells after stimulation of PBMCs with overlapping peptide pools. After thawing, the PBMCs (5 × 10^6^ cells/ml) were stimulated with 0.6 nmol/ml PepTivator SARS-CoV-2 Select-premium grade (Miltenyi Biotec, Bergisch Gladbach, Germany) for 16–18 h, in the presence of 10 μg/ml anti-human CD28/CD49d Abs (Thermo Fisher Scientific) for co-stimulation. A CEF peptide pool (4 μg/ml; Mabtech AB, Hamburg, Germany), composed of well-defined peptides derived from cytomegalovirus (c), Epstein–Barr virus (e), and influenza virus (f), was used as the positive control (16) and DMSO was used as the negative control. A fluorescein isothiocyanate (FITC)–anti-human CD4 Ab (clone, RPA-T4; BD Biosciences) was applied concomitantly with antigen stimulation for staining. After the antigen stimulation, dead cells were stained with Fixable Viability Dye eFluor 506 (Thermo Fisher Scientific). Surface antigens were stained with BUV496-anti-human CD8 (clone, RPA-T8), BUV395-anti-human CD137 (clone, 4B4-1), phycoerythrin (PE)-CF594-anti-human OX40 (clone, ACT35), and PE-anti-human CD69 (clone, FN50) Abs (all from BD Biosciences). Brilliant Stain Buffer (BD Biosciences) was added to each sample. Stained PBMCs were analyzed using an LSR II flow cytometer (BD Biosciences) with a minimum target event count of 500,000 cells. Data were analyzed using FlowJo software (version 10.7.1; BD Biosciences).

The frequencies of SARS-CoV-2-specific T cells (AIM^+^ T cells; OX40^+^ CD137^+^ CD4^+^ T cells or CD69^+^ CD137^+^ CD8^+^ T cells) (28) were evaluated. The percentages of target populations in the unstimulated specimens (DMSO control) were subtracted from those in the antigen-stimulated specimens to account for a nonspecific response (19).

### Detection of SARS-CoV-2 Antigen-Specific Memory B Cells

To measure SARS-CoV-2-specific memory B cells, biotinylated protein antigens were individually multimerized with allophycocyanin (APC)-conjugated streptavidin (SA-APC; Thermo Fisher Scientific) at 4°C for 1 h. Biotinylation was performed using the EZ-Link™ Sulfo-NHS-Biotinylation Kit (Thermo Fisher Scientific) following the standard protocol of the manufacturer. To make a complex of RBD + ACE2, RBD proteins were incubated with ACE2 (1:1 molar ratio) at 4°C for 1 h. Biotinylated spike proteins were multimerized with SA-APC at a 3.4:1 ratio, biotinylated RBD at a 1.4:1 ratio, biotinylated RBD-ACE2 complex at a 3.85:1 ratio, and biotinylated at a 1.25:1 ratio. SA-APC was used as a decoy probe, negative control, to gate out SARS-CoV-2-nonspecific streptavidin-binding B cells.

After thawing, the PBMCs (3 × 10^6^ cells/ml) were stained with 40 nM antigen probe (Spike, RBD, RBD + ACE2, and NC), and BUV395-anti-human CD19 (clone, SJ25-C1), BV421-anti-human CD27 (clone, M-T271), BV605-anti-human IgM (clone, G20-127), FITC-anti-human IgA (polyclonal), Alexa Fluor^®^ 700-anti-human IgG (clone, G18-145), and PE-anti-human IgD (clone, IA6-2) Abs (all from BD Biosciences, except for FITC-anti-IgA Ab, from Thermo Fisher Scientific). Brilliant Stain Buffer (BD Biosciences) was added to each sample. Stained PBMCs were acquired using an LSR II flow cytometer (BD Biosciences) with a minimum target event count of 200,000 cells and analyzed using FlowJo software (version 10.7.1; BD Biosciences).

### Statistical Analyses

The Kruskal–Wallis rank-sum test or linear-by-linear association was performed to compare continuous and categorical clinical characteristics, respectively, among the asymptomatic, mild, and severe patients. Data are expressed as mean ± standard errors of the mean (SEM) and as dot plots. The Kruskal–Wallis rank-sum test with Dunn’s *post hoc* test for multiple comparisons was used to compare the binding activity of Abs, phagocytic capability of Abs, and frequencies of activated T cells upon antigenic stimulation and antigen-specific memory B cells according to disease severity. An unpaired two-tailed Student’s *t*-test was also used to compare the binding capacity of Abs. The coefficient of determination (R^2^) was calculated by linear regression analysis between 8 and 12 months post-COVID-19.

*P <*0.05 was considered statistically significant. All statistical analyses were two-tailed and performed using PASW for Windows (version 25.0; IBM Corp., Armonk, NY, USA) or GraphPad Prism 9 (GraphPad Software, La Jolla, CA, USA). All graphs were generated using Prism 9.

### High Dimensional Analysis

All data were analyzed using custom R scripts (R Foundation for Statistical Computing, Vienna, Austria. Forty-seven parameters were selected, namely, Ab, phagocytic capability, and cell frequency data; participants with missing values were excluded. Since each participant has multiple parameters from a different experiment, the parameters should be reduced to interpret relationship among participants. We adopted the principal component analysis (PCA), a technique for stepping down the dimension of a dataset by combining the parameters with maximum variance, called PC, to determine the relationship among participants. Thus, we projected the dataset combined with multiple parameters onto the two-dimensional space by sequentially orthogonal transforming the eigenvalues (i.e., PC) of the uncorrelated low-dimensional spaces on each axis. The Kruskal–Wallis rank-sum test with Dunn’s *post hoc* test for multiple comparisons was used to compare PC scores according to disease severity. To discover parameters related to PC-1 or PC-2 axis in a quantitative approach, we assumed that the axis-related parameters were significantly correlated with the axes in PC space. Spearman correlation was used for comparing the PC coordinates and levels of the 47 parameters using the cor. test function in R with the “Spearman” option. The level of significance was adjusted using Bonferroni correction for multiple hypothesis testing. The heatmap data are Spearman’s rank correlation coefficient (*ρ*) and adjusted *P*-values for each feature versus PC-1 and PC-2, for all participants and for selected participant groups compared to the severe group. Thus, the PCA was used to determine factors exerting a major influence on the progression of COVID-19. Source codes and data files are available from the authors upon request.

## Results

### Study Participants

A total of 24 patients (7, 9, and 8 patients who had asymptomatic, mild, and severe COVID-19, respectively) were included in this study (16). Among them, 7, 2, and 7 patients and 6, 9, and 8 patients in these three groups were available for analyses at 8 months and 1 year post-COVID-19, respectively. The clinical characteristics and detailed information of each patient are shown in **Table 1** and **Supplementary Table S1**, respectively.

**Table 1:** Clinical characteristics of patients included in analyses for 1 year post-COVID-19.

	HC (2021) (*n* = 8)	Completely asymptomatic (*n* = 7)	Mild (*n* = 9)	Severe (*n* = 8)	*P* ^a^
Age, median years (range)	39 (22–50)	25 (20–28)	53 (24–72)	63 (39–76)	0.001
Male gender, *n* (%)	4 (50.0)	5 (71.4)	3 (33.3)	6 (75.0)	0.831
Underlying diseases, *n* (%)					
Diabetes mellitus	0 (0)	0 (0)	3 (33.3)	3 (37.5)	0.108
Hypertension	0 (0)	0 (0)	2 (22.2)	5 (62.5)	0.009
Maximal O_2_ demand	NA	No	No	HFNC (NP to MV)	NA
Treatment, *n* (%)					
Remdesivir	NA	0 (0)	1 (11.1)	7 (87.5)	<0.001
Baricitinib	NA	0 (0)	1 (11.1)	3 (37.5)	0.054
Steroid	NA	0 (0)	0 (0)	2 (25.0)	0.079
Days of sample collection from the onset of COVID-19, median (range)					
For 8 months	NA	231 (229–235)	237 (192–265)	197 (185–220)	0.003
For 12 months	NA	351 (343–355)^b^	370 (315–383)	356 (318–368)	0.405

HC, healthy control; NA, not applicable; HFNC, high flow nasal canula; NP, nasal prong; MV, mechanical ventilation.

a Healthy control group was not included in the P-value calculation.

b One patient was unavailable at 12-months post COVID-19.

No patient had a history of immunodeficiency or re-exposure to COVID-19 during the follow-up period. The median (range) ages of the asymptomatic, mild, and severe patients were 25 (20–28), 53 (24–72), and 63 (39–76) years, respectively (*P* = 0.001). One mild and three severe patients received baricitinib and two severe patients received therapeutic doses of steroids during hospitalization.

Eight healthy volunteers were included in the HC (2021) group. Their median (range) age was 39 (22–50) years, and four (50.0%) were male.

### Proportional Ab Responses According to Disease Severity

**Figure 1:**
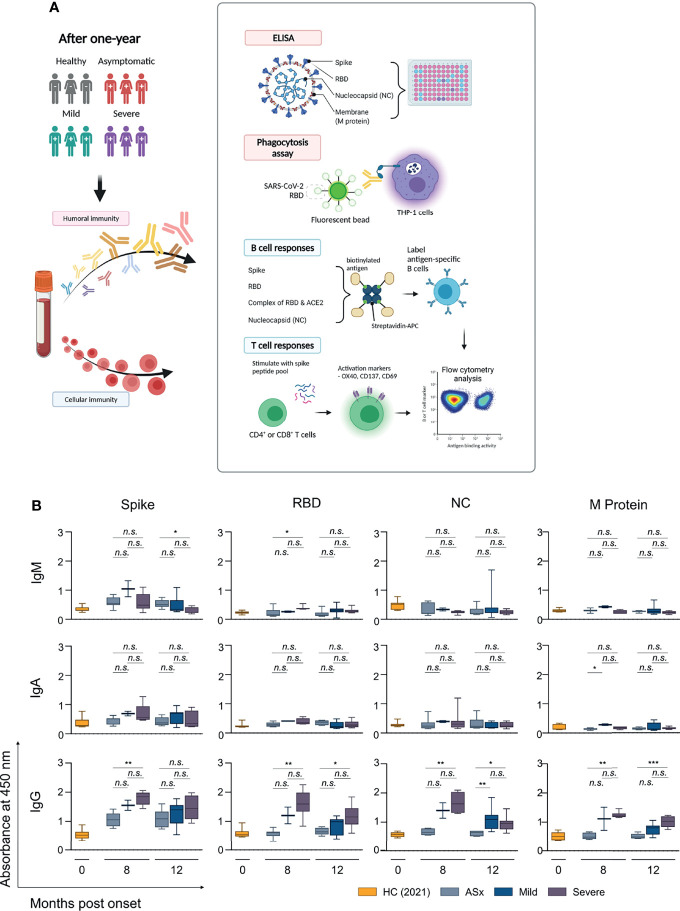
Longitudinal analysis of anti-SARS-CoV-2 antibody in COVID-19 patients over time according to the severity of illness. **(A)** Schematic illustration (created with BioRender.com) of experimental design for analyzing humoral and cellular immune response to SARS-CoV-2 according to the disease severity, including **Figures 1**–**4**. **(B)** Binding activities of each Ig isotype to SARS-CoV-2 proteins. Diluted plasma samples (1:100) in PBS were incubated with SARS-CoV-2 spike protein, receptor-binding domain (RBD), nucleocapsid (NC), and M protein. Corresponding isotyping Abs determined binding activities of IgM, IgA, and IgG to SARS-CoV-2 antigens. ASx, asymptomatic (8-month: *n* = 7, 12-month: *n* = 6), Mild (8-month: *n* = 2, 12-month: *n* = 9), Severe (8-month: *n* = 7, 12-month: *n* = 8). Statistical analyses were performed using the Kruskal–Wallis rank-sum test with Dunn’s *post hoc* test in GraphPad Prism (*n.s*.: *P > *0.05, *P < 0.05, **P < 0.01, ***P < 0.001).

To assess the dynamics of Ab titers against SARS-CoV-2 infection over time, plasma specimens of COVID-19 patients were collected 8 and 12 months after symptom onset. The dynamics of IgM, IgA, IgG, and four IgG subclasses against four SARS-CoV-2 specific proteins were measured using an in-house enzyme-linked immunosorbent assay (ELISA) with a 1:100 titer (**Figure 1** and **Supplementary Figures S1–3**).

First, we analyzed the correlation between Ab responses and the severity of COVID-19. The circulating IgG with binding reactivity to SARS-CoV-2 spike, RBD, NC, and M proteins was positively correlated with the severity of past COVID-19 (**Supplementary Figure S1**, right column). With the exception of the M protein, circulating IgA reactivities to SARS-CoV-2 spike, RBD, and NC proteins positively correlated with the severity of COVID-19 in 8-month samples, but not in 12-month samples, and the overall binding response of IgA was weaker than that of IgG (**Supplementary Figure S1**, middle column). In addition, IgG showed a positive correlation in both 8 and 12-month samples, but IgA did not show any detectable reactivity to four SARS-CoV-2 proteins in 12-month samples (**Figure 1** and **Supplementary Figure S1**, middle lane). However, IgM did not show a significant binding response neither a positive correlation with the severity of COVID-19. In general, most IgM plasma cells were short-lived, especially compared to IgG plasma cells. Therefore, the Ab titer of antigen-specific IgM, unlike IgM^+^ antigen-specific memory B cells, decreases over time after infection [reviewed in (29)].

The circulating IgG at 8 months in asymptomatic patients showed the binding signals for only spike protein, and plasma IgM also showed weak binding signals against spike protein (**Figure 1** and **Supplementary Figures S2, S3**). A major subclass of anti-spike IgG in asymptomatic patients was IgG_1_ (**Supplementary Figure S2**). Interestingly, the results indicated that most anti-spike IgM and IgG may not inhibit the interaction between ACE2 and spike protein because the circulating Abs did not show binding against RBD protein (**Figure 1** and **Supplementary Figure S2**).

In addition, both mild and severe patients showed significant Ab responses for NC and M protein, although these responses weakened over time. A major isotype of SARS-CoV-2-specific Abs was IgG in both mild and severe patients, and severe patients showed higher Ab responses to four SARS-CoV-2 proteins than mild patients. In particular, anti-NC Abs in severe patients were mainly composed of IgG_1_ and IgG_2_ (**Supplementary Figure S2**).

In summary, three noteworthy results emerged from the analysis of humoral immunity. First, overall Ab responses were proportional to the severity of COVID-19, as previously reported (30–32). Second, most anti-spike Abs in asymptomatic patients were non-RBD binding Abs, but severe patients had high levels of RBD-binding Abs. Third, mild and severe patients had the detectable circulating Abs for four SARS-CoV-2 proteins, in contrast to asymptomatic patients.

### Antibody-Dependent Cellular Phagocytosis (ADCP) According to Disease Severity

**Figure 2:**
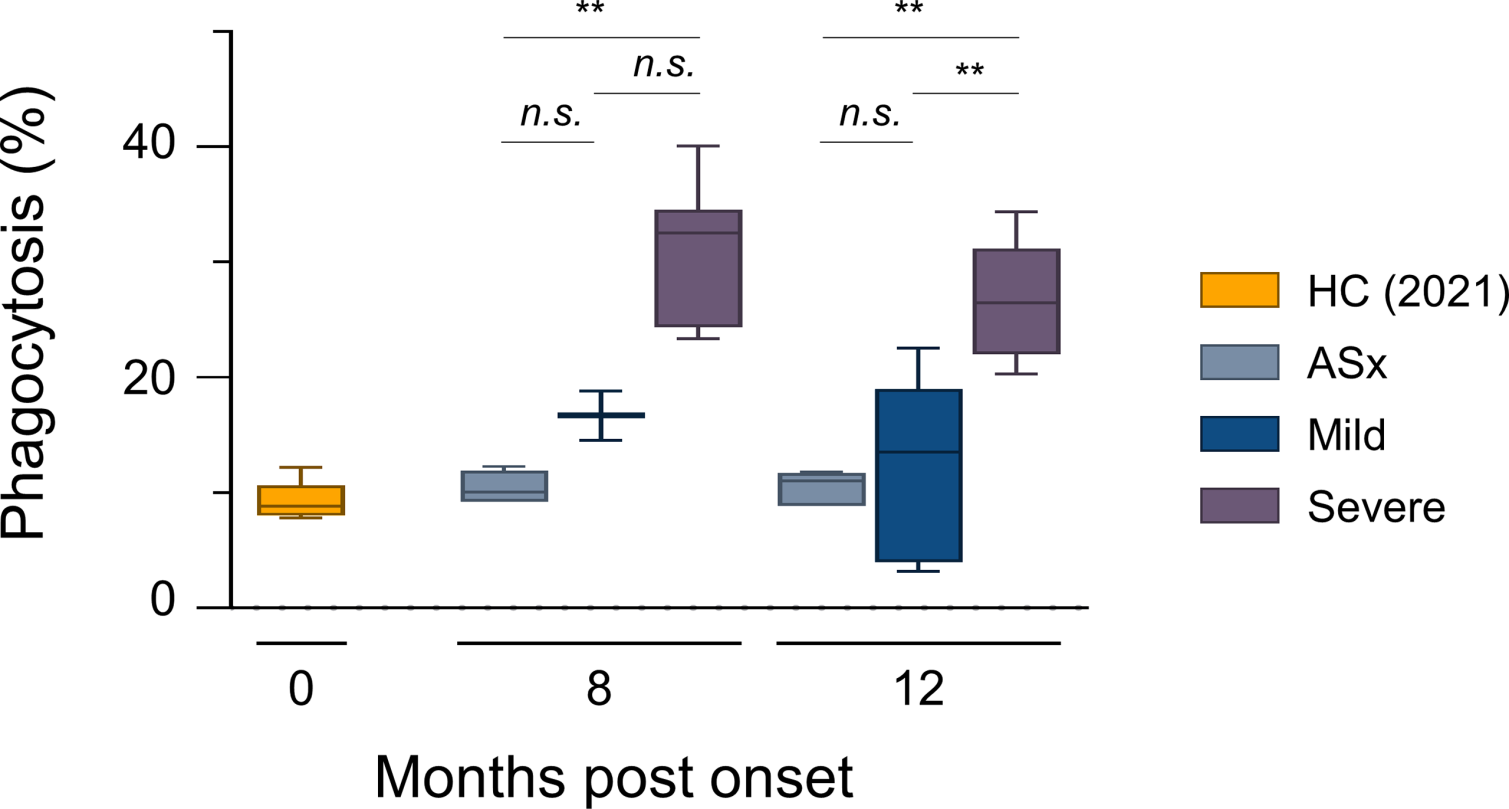
*In vitro* phagocytic capability assays according to the severity of illness. ADCP assays with THP-1 cells as effectors and RBD-coated fluorescent, carboxylate-modified 1 μm red (580/605 nm, F8821) bead as targets (effector: target ratio of 10:1). RBD-coated red beads were pre-incubated with the diluted plasma for 20 min and then incubated with THP-1 cells for 2 h. Phagocytic activities were determined by flow cytometric analysis. % of Phagocytosis = numbers of red (580/605 nm) − positive THP-1 cells/numbers of total THP-1 cells × 100. Results are representative data from three independent experiments. Statistical analysis was performed using the Kruskal–Wallis rank-sum test with Dunn’s *post hoc* test in GraphPad Prism (*n.s*.: *P > *0.05, **P < 0.01).

Next, we examined the phagocytic capability of circulating Abs using THP-1 cells. RBD-coated green -> red fluorescent beads were incubated with plasma for Ab-mediated opsonization, followed by incubation with THP-1 cells for 2 h at 37°C, as previously reported (26, 33). THP-1 cells show low FcγRI expression, and high FcγRIIa and FcαRI expression, where these are regarded as the major scavenger receptors for ADCP activity (26, 34). Thus, for ADCP, the concentration and affinity of IgA and IgG against antigens are critical, and the phagocytic ability of Abs was shown in proportion to the IgA and IgG levels of each patient (**Supplementary Figures S1, S3**). As expected, the phagocytic capability of Abs was highest in severe patients, but interestingly, there was no significant difference in the phagocytic capacity of circulating Abs between mild and asymptomatic patients (**Figure 2**).

### Memory B-Cell Responses to SARS-CoV-2 According to Disease Severity

To assess the memory B-cell responses to SARS-CoV-2 for comparison with the Ab responses by long-lived plasma cells, we assessed the SARS-CoV-2-specific memory B cells by staining PBMC APC-multimeric SARS-CoV-2 antigens (spike, RBD, complex of RBD + ACE2, and NC) together with isotype-specific Abs (anti-IgM, anti-IgA, and anti-IgG Abs) (**Supplementary Figure S4**).

**Figure 3:**
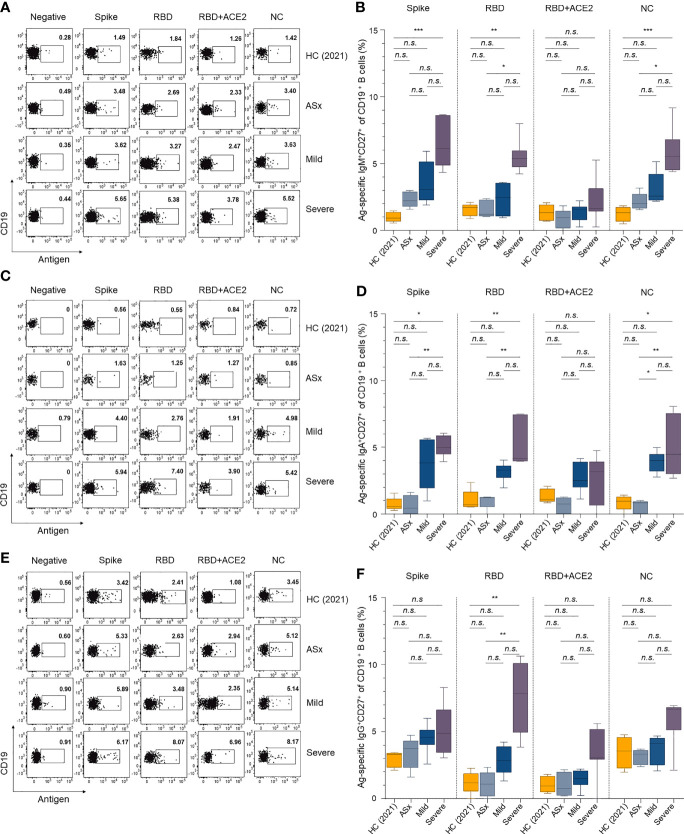
Memory B-cell responses against SARS-CoV-2 according to the severity of illness. **(A, C, E)** Representative gating strategy for IgM^+^, IgA^+^, and IgG^+^ antigen-specific memory (CD27^+^ CD19^+^) B cells. **(B, D, F)** Frequencies of IgM^+^, IgA^+^, and IgG^+^ antigen-specific memory B cells according to the severity of illness and different antigens. Statistical analysis was performed using the Kruskal–Wallis rank-sum test with Dunn’s *post hoc* test in GraphPad Prism (*n.s*.: *P > *0.05, *P < 0.05, **P < 0.01, ***P < 0.001).

Four noteworthy results emerged. First, the percentages of all SARS-CoV-2 antigen-specific memory B cells were proportional to the severity of COVID-19, and the severe group consistently showed higher proportions of all IgM^+^, IgA^+^ and IgG^+^ memory B cells compared to the other groups. The abundance of memory B cells according to Ab isotype (IgM, IgA, IgG, and IgD) was in the order of IgG > IgA > IgM (**Figure 3**), similar to the circulating responses (**Figure 1**). Second, the percentages of spike-specific memory B cells were similar to those of NC-specific memory B cells. This was seen in all severity groups and Ab isotypes (**Figure 3**). In addition, the severe group showed a higher frequency of IgM^+^ NC-specific memory B cells compared to the asymptomatic and mild groups, but the frequencies of IgM^+^ spike- and NC-specific memory B cells were otherwise equivalent (**Figures 3A**). For IgA^+^ spike- RBD, RBD + ACE2, and NC-specific memory B cells, the mild and severe groups showed higher frequencies than the asymptomatic and HC (2021) groups, and both showed equal levels (**Figures 3C**). For IgG^+^ spike- and NC-specific memory B cells, the mild and severe groups showed similar frequencies, and the asymptomatic group had equivalent levels with the HC (2021) group (**Figures 3E**). Third, the asymptomatic group had fewer neutralizing B cell receptors (BCRs) than the mild and severe groups (**Figures 3B, D**). However, there were no statistical differences in the frequency of memory B cells with (presumed to be) non-neutralizing (complex of RBD + ACE2 reactive Abs) BCRs according to severity, although their average frequency in the mild and severe groups was higher than in the asymptomatic and HC (2021) groups. In the asymptomatic and HC (2021) groups, the percentage of IgG^+^ RBD-specific memory B cells (mean ± SEM, 1.21 ± 0.37% for HC (2021), 1.07 ± 0.41% for ASx) was similar to that of complex of RBD + ACE2-specific memory B cells (1.06 ± 0.27% for HC (2021), 1.05 ± 0.40% for ASx). However, the mild group showed a slightly higher percentage of RBD-specific IgG^+^ memory B cells (2.88 ± 0.44%) than that of complex of IgG^+^ RBD + ACE2-specific memory B cells (1.47 ± 0.29%). Moreover, the difference between them was significantly larger in the severe group (7.49 ± 0.95% for RBD and 3.34 ± 0.69% for the complex of RBD + ACE2). This was consistent with the IgA^+^ B-cell responses (**Figure 3**) and suggests that severe patients have a longer memory B cell response for neutralizing BCRs against SARS-CoV-2, where this memory B cell response was proportional to the severity of COVID-19. Finally, for non-class-switched IgD^+^ memory B cells, memory responses to spike, RBD, and NC proteins were also generated in patients with COVID-19 (**Supplementary Figure S5**).

Both circulating Ab and memory B-cell responses showed a positive correlation with the severity of COVID-19. In addition, the mild and severe groups showed stronger RBD reactivity and ACE2 competitive binding than asymptomatic patients. This suggests that memory B cells had more neutralizing activity in the mild and severe groups than the asymptomatic group.

### Memory T-Cell Response to SARS-CoV-2 According to Disease Severity

**Figure 4:**
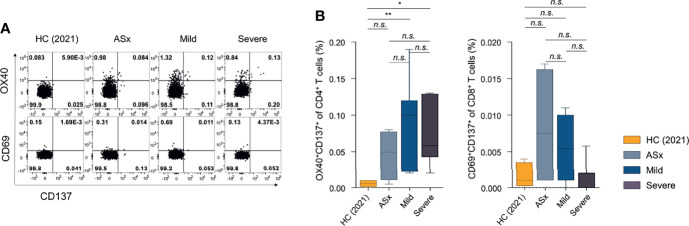
Frequencies of SARS-CoV-2-specific activation-induced markers (AIM)^+^ CD4^+^ or CD8^+^ T cells according to the severity of illness. **(A)** Frequencies of SARS-CoV-2-specific AIM^+^ (OX40^+^CD137^+^) CD4^+^ T cells according to the severity of illness. **(B)** Frequencies of SARS-CoV-2-specific AIM^+^ (CD69^+^CD137^+^) CD8^+^ T cells according to the severity of illness. Statistical analyses were performed using the Kruskal-Wallis rank-sum test with Dunn’s post hoc test in GraphPad Prism (*n.s*. : P > 0.05, *P < 0.05, **P < 0.01).

The frequencies of SARS-CoV-2-specific CD4^+^ (OX40^+^CD137^+^) T cells were significantly higher in mild and severe patients than healthy controls (**Figure 4**). However, SARS-CoV-2-specific CD8^+^ (CD69^+^CD137^+^) T-cell responses were not statistically different from those of healthy controls. Memory T-cell responses at 1 year post-COVID-19 were most evident in CD4^+^ T cells in symptomatic patients, consistent with previous reports of memory T-cell responses at 8 months post-symptom onset (23).

### Integrated Analysis of Immune Responses to SARS-CoV-2 According to Disease Severity

**Figure 5:**
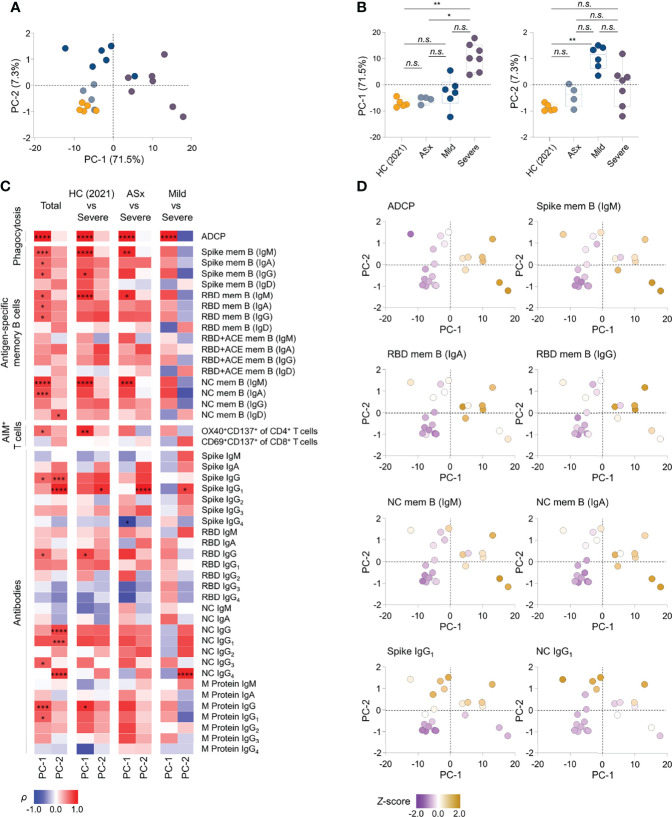
Integrated analysis of immune components to SARS-CoV-2 according to the severity. **(A)** Principal component analysis (PCA) of aggregated immune responses data against SARS-CoV-2, namely, circulating Abs against SARS-CoV-2 proteins, the Ab-dependent phagocytic capability of plasma, and frequencies of antigen-specific memory cells over disease severity of illness from PBMCs collected at 12 months in patients and healthy subject. HC (2021): healthy control (*n* = 5), ASx: asymptomatic (*n* = 4), Mild (*n* = 6), Severe (*n* = 7). **(B)** Summary boxplots of PC-1 and PC-2 scores over disease severity of illness. Statistics were calculated using non-parametric ANOVA with correction for multiple comparisons using statistical hypothesis testing (Dunn’s test) (*n.s*.: *P > *0.05, *P < 0.05, **P < 0.01, ***P < 0.001). Boxplots represent median with interquartile range. **(C)** Correlations of antigen-specific immune responses against the PC-1 and PC-2, displaying contributions of 47 immune components to PC-1 and PC-2 in group comparisons. The color represents Spearman’s rank correlation coefficient (*ρ*); red color indicates positive correlations, and blue indicates negative correlations among patient groups of disease severity. Statistics were adjusted using Bonferroni correction in the consideration of multiple statistical hypothesis n.s.: adj. P >0.05, * adj. P < 0.05, ** adj. P < 0.01, *** adj. P < 0.005, **** adj. P < 0.001. **(D)** Representative scatter plots displaying the relative levels of selected features onto PC space. The color represents *z*-score of each feature according to heat scale ranges from purple (low) to yellowish brown (high) color. PC-1, principal component-1; PC-2, principal component-2; mem B, memory B cells.

To investigate immune components explaining variation in disease severity, we performed PCA using 47 integrated parameters, namely, Abs against SARS-CoV-2 antigens, phagocytic capability of Ab, and frequencies of antigen-specific memory cells from 22 selected participants. In PC space, mild and severe groups were discriminated from healthy and asymptomatic groups, indicating that the 47 immune responses are valuable for investigating differences in severity (**Figure 5**). Interestingly, the severe and mild groups were mainly distributed over the PC-1 and PC-2 axes, respectively (**Figure 5**), suggesting that the features defining PC-1 and PC-2 could be the main immune responses of the mild and severe groups.

Next, we determined the contribution of all parameters (immune responses) to PC-1 and PC-2 in all group comparisons, and then calculated the Spearman’s correlation coefficient (*ρ*) for all samples to identify significant features (adjusted *P <*0.05) reflecting PC-1 and PC-2 (**Figure 5**). Further, to select features with consistent correlation trends regardless of disease group, we performed additional correlation analysis between the features and PC axes using selected participants from the groups, filtering out features out of the detection range. Nine features were found to represent PC-1, namely, ADCP, IgM^+^, and IgA^+^ spike- and NC-specific memory B cells [spike mem B (IgM, IgA), NC mem B (IgM, IgA)], IgM^+^, IgA^+^ and IgG^+^ RBD-specific memory B cells [(RBD mem B (IgM, IgA, IgG)], and anti-RBD IgG. Three features were found to be associated with PC-2, namely, anti-NC IgG_1_, anti-spike IgG_1_, and IgD^+^ RBD-specific memory B cells [RBD mem B (IgD)]. In addition, the majority of the selected features (7 of 9) correlated with PC-1 are antigen-specific memory B cells, and the features correlated with PC-2were Abs (2 of 3 features). Interestingly, none of the features satisfied the criteria for both PC-1 and PC-2 (**Figure 5**), suggesting that mild and severe patients display distinct immune responses.

In summary, this unbiased integrated analysis of 47 parameters of immune responses against SARS-CoV-2 enabled us to identify key features distinguishing mild and severe disease groups from healthy and asymptomatic groups. The overall responses of memory B cells and phagocytic capability of Abs correlated with the severity of SARS-CoV-2 infection, indicating that an integrated analysis of the humoral immune response-related features can determine the immunological characteristics of severe disease. Patients with severe COVID-19 had increased expression of spike- and NC-specific memory B cells, which play a crucial role upon SARS-CoV-2 reinfection, whereas anti-NC IgG_1_ and anti-spike IgG_1_ were abundant in patients with mild COVID-19, enabling discrimination based on disease severity.

## Discussion

In this study, we evaluated humoral and cellular immune responses 1 year post-COVID-19, in terms of the binding activities of circulating Abs to SARS-CoV-2-specific proteins, phagocytic capability of circulating Abs, and memory B- and CD4^+^ T-cell responses. We firstly showed that the breadth and functionality of serologic and cellular responses, particularly phagocytic capacity of antibodies and memory B-cell responses, were dependent on the severity of the prior infection, even at 1 year post-COVID-19.

Although we only measured circulating Ab levels, and not respiratory mucosal Abs, there were several key findings. It is very important to determine how long a convalescent patient maintains a protective immune response to SARS-CoV-2, because this has practical significance for optimal vaccination timing in those who have had a natural infection with SARS-CoV-2, and explain the seemingly higher rate of SARS-CoV-2 reinfection in those who had mild or asymptomatic compared to severe illness (12–14). Although the level of neutralizing Ab, a potential correlate of protection (COP) (30), is known to be higher in severe than in mild or asymptomatic patients for up to 1 year post-COVID-19 (35), our study yielded several additional findings suggesting differences in the level of protective immunity according to the disease severity.

First, humoral and cellular responses showed a strong positive correlation with the severity of past COVID-19 (**Figures 1**, **3** and **Supplementary Figure S1**) (36) and the severe group had a higher proportion of ACE2-competitively RBD-binding memory B cells than the asymptomatic and mild groups (**Figure 3**), although the exact reason has not been clarified yet. Meanwhile, the higher levels of SARS-CoV-2 antigen-specific Ab titers in severe patients (**Figure 1**) are possibly due to a higher viral load (37) or excessive activation of T cells (23, 38).

Second, the severe group still has ADCP capacity maintained by circulating Abs, in contrast to the mild and asymptomatic groups (**Figure 2**). Our results showed that the severe group had strong anti-SARS-CoV-2 IgA and IgG responses, which can activate scavenger receptors, such as Fcγ and Fcα receptors (39), more strongly during both humoral and cellular responses compared to mild and asymptomatic groups (**Figures 1**, **3**). Recent studies have reported that neutralizing Abs can prevent reinfection, and non-neutralizing Abs do not prevent reinfection [*reviewed in* (40)]. Still, they can reduce the severity by clearing infected cells through Fc-mediated effector functions in the animal model (41). Our results also showed that plasma Abs in the severe group were sufficient to do phagocytosis, and this result may suggest the possibility of milder course of reinfection in the severe patients.

Third, the severe group showed higher anti-NC Ab levels than the other severity groups. In fact, complement hyperactivation by NC is a key feature of severe COVID-19 (42). It is implicated in its pathogenesis (43, 44), but a correlation between anti-SARS-CoV-2 Abs and complement hyperactivation has not been clearly identified (45, 46). A recent report suggested that anti-NC Abs are capable of suppressing NC-mediated complement hyperactivation (46). In our study, severe patients maintained strong humoral and cellular immune responses against NC compared to the other groups, which may explain the very low reinfection rate in the severe group. Although these Abs have not yet been extensively studied as COP candidates, they are all highly likely to protect against SARS-CoV-2 reinfection, or at least to successfully control the virus during the early stage of the disease (47, 48), and merit further evaluation.

Fourth, defective follicular helper T-cell differentiation and humoral immune-induced dysregulation are known to occur in the early stage of COVID-19 (49). The result of this study enhances our understanding that the higher long-term memory B-cell response in severe patients than in mild ones is restored by the germinal center response, albeit later, that occurs after the extrafollicular response (29).

Overall, these features of humoral and cellular immune responses could provide a clue for tailored vaccination of COVID-19 patients. COVID-19 vaccination is currently recommended in patients with a history of natural infection with SARS-CoV-2 (50). Since it has been reported that broader and stronger immune responses could be achieved by vaccination of those who have had COVID-19, which might be particularly helpful in the era of variants (51, 52), vaccination of such patients is widely accepted. However, there is a still paucity of data on the timing and number of doses that should be given to those previously infected with SARS-CoV-2 (50). Our study showed that immune responses at 1 year post-COVID-19 differed according to the severity of the original illness, which indicates that vaccination strategies should prioritize certain groups and adjust the timing of vaccination post-COVID-19, for example. Patients who had asymptomatic or mild illness might be vaccinated earlier than severe patients, since asymptomatic and mild patients exhibit lower levels of humoral protection and memory responses against SARS-CoV-2 than severe patients at 1 year post-COVID-19.

There are several limitations in the present study. First, since we could not follow up immune responses from the right after the infection, it was difficult to assess longitudinal or relative lasting immunity. Second, the samples for 8 months post-COVID-19 were collected significantly earlier in severe patients than in mild or asymptomatic ones. However, the samples were drawn in similar time points for 1 year post-COVID-19, which we would like to focus on our study. Third, because the age distribution was not identical for each group, the values from healthy control group should be interpreted cautiously.

In conclusion, we observed significant humoral and cellular immune responses against SARS-CoV-2 at 1 year post-COVID-19, in particular the phagocytic capacity of circulating Abs and memory B-cell responses; the magnitude of these responses was greater in severe than mild and asymptomatic patients. To enhance our understanding of COVID-19 and improve vaccination strategies, further studies on these immune responses against SARS-CoV-2 are warranted.

## Data Availability Statement

The original contributions presented in the study are included in the article/**Supplementary Material**. Further inquiries can be directed to the corresponding authors.

## Ethics Statement

The studies involving human participants were reviewed and approved by the Institutional Review Board of Seoul National University Hospital (IRB No. H-2004-158-1118). The patients/participants provided their written informed consent to participate in this study.

## Author Contributions

CKK, C-HL, WBP, and H-RK conceived and designed the project. CKK, MK, JH, GK, ISK, C-HL, WBP, and H-RK analyzed the data. CKK, EC, PGC, NJK, WBP, and M-dO collected the human PBMCs, CKK, MK, GK, SL, ISK, D-SL, HMS, Y-WK, C-HL, and H-RK performed flow cytometric analysis. CKK, MK, JH, J-YS, DS, C-HL, and H-RK performed serologic analysis. CKK, MK, JH, C-HL, WBP, and H-RK wrote the manuscript with the help from all authors. C-HL, WBP, and H-RK had full access to all data in the study and took responsibility for the integrity of the data, and also for the manuscript. All authors listed have made a substantial, direct, and intellectual contribution to the work and approved it for publication.

## Funding

This work was supported in part by the Creative-Pioneering Researchers Program through Seoul National University (to H-RK); the Bio & Medical Technology Development Program of the National Research Foundation (NRF) & funded by the Korean government (MSIT) (2018M3A9H4055197, to H-RK and 2021M3A9I2080496, to H-RK and WP), the New Faculty Startup Fund from Seoul National University (to C-HL); and the Seoul National University Hospital Research Fund (03-2021-0090, to N-JK).

## Conflict of Interest

The authors declare that the research was conducted in the absence of any commercial or financial relationships that could be construed as a potential conflict of interest.

## Publisher’s Note

All claims expressed in this article are solely those of the authors and do not necessarily represent those of their affiliated organizations, or those of the publisher, the editors and the reviewers. Any product that may be evaluated in this article, or claim that may be made by its manufacturer, is not guaranteed or endorsed by the publisher.

## Supplementary Material

Supplementary Figure S1 Correlation of antibody response and severity of COVID-19 patients. Each antibody’s relative binding activity to SARS-CoV-2-specific protein was presented with severity of COVID-19 patients. Coefficient of determination (R2) was calculated by linear regression analysis. ASx: asymptomatic (8-month: n = 7, 12-month: n = 6), Mild (8-month: n = 2, 12-month: n = 9), Severe (8-month: n = 7, 12-month: n = 8).

Supplementary Figure S2 Binding activities of four IgG subclasses to SARS-CoV-2 proteins. Diluted plasma samples (1:100) in PBS were incubated with SARS-CoV-2 spike, RBD, NC and M protein. Binding activities of IgM, IgA, and IgG to SARS-CoV-2 antigens were determined by proper Abs. HC (2021): healthy control (n = 8), ASx: asymptomatic (8-month: n = 7, 12-month: n = 6), Mild (8-month: n = 2, 12-month: n = 9), Severe (8-month: n = 7, 12-month: n = 8). Statistical analyses were performed using the Kruskal-Wallis rank-sum test with Dunn’s post hoc test in GraphPad Prism (n.s.: P > 0.05, *P < 0.05, **P < 0.01, ***P < 0.001).

Supplementary Figure S3 Relative binding activities to each antigen of anti-SARS-CoV-2 antibody in COVID-19 patients over time. Antibody binding capacity to each antigen shown in **Figure 1** was normalized based on the value for HC. HC (2021): healthy control (n = 8), ASx: asymptomatic (8-month: n = 7, 12-month: n = 6), Mild (8-month: n = 2, 12-month: n = 9), Severe (8-month: n = 7, 12-month: n = 8).

Supplementary Figure S4 Representative gating strategy used for flow cytometric analysis for memory B-cell response. Gating strategy to analyze viable IgG+, IgA+, IgM+, and IgD+ memory B cells.

Supplementary Figure S5 Frequencies of IgD+ antigen-specific memory B cells according to the severity of illness. A Representative gating strategy for IgD+ antigen-specific memory B cells. B Frequencies of IgD+ antigen-specific memory B cells according to the severity of illness and different antigens.

Supplementary Figure S6 Representative gating strategy used for flow cytometric analysis for memory T-cell response. Gating strategy to analyze viable OX40+CD137+ CD4+ T cells and CD69+CD137+ CD8 T+ cells.

## References

[1] Korber B Fischer WM Gnanakaran S Yoon H Theiler J Abfalterer W. Tracking Changes in SARS-CoV-2 Spike: Evidence That D614G Increases Infectivity of the COVID-19 Virus. Cell (2020) 182(4):812–27 e19. doi: 10.1016/j.cell.2020.06.043 32697968 PMC7332439

[2] Planas D Bruel T Grzelak L Guivel-Benhassine F Staropoli I Porrot F. Sensitivity of Infectious SARS-CoV-2 B.1.1.7 and B.1.351 Variants to Neutralizing Antibodies. Nat Med (2021) 27(5):917–24. doi: 10.1038/s41591-021-01318-5 33772244

[3] Singh J Rahman SA Ehtesham NZ Hira S Hasnain SE. SARS-CoV-2 Variants of Concern are Emerging in India. Nat Med (2021) 27(7):1131–3. doi: 10.1038/s41591-021-01397-4 34045737

[4] Haas EJ Angulo FJ McLaughlin JM Anis E Singer SR Khan F. Impact and Effectiveness of mRNA BNT162b2 Vaccine Against SARS-CoV-2 Infections and COVID-19 Cases, Hospitalisations, and Deaths Following a Nationwide Vaccination Campaign in Israel: An Observational Study Using National Surveillance Data. Lancet (2021) 397(10287):1819–29. doi: 10.1016/S0140-6736(21)00947-8 PMC8099315 33964222

[5] Abu-Raddad LJ Chemaitelly H Butt AA National Study Group for C-V. Effectiveness of the BNT162b2 Covid-19 Vaccine Against the B.1.1.7 and B.1.351 Variants. N Engl J Med (2021) 385(2):187–9. doi: 10.1056/NEJMc2104974 PMC8117967 33951357

[6] Lopez Bernal J Andrews N Gower C Gallagher E Simmons R Thelwall S. Effectiveness of Covid-19 Vaccines Against the B.1.617.2 (Delta) Variant. N Engl J Med (2021) 385(7):585–94. doi: 10.1056/NEJMoa2108891 PMC8314739 34289274

[7] Acuna-Zegarra MA Diaz-Infante S Baca-Carrasco D Olmos-Liceaga D. COVID-19 Optimal Vaccination Policies: A Modeling Study on Efficacy, Natural and Vaccine-Induced Immunity Responses. Math Biosci (2021) 337:108614. doi: 10.1016/j.mbs.2021.108614 33961878 PMC8095066

[8] Hansen CH Michlmayr D Gubbels SM Molbak K Ethelberg S. Assessment of Protection Against Reinfection With SARS-CoV-2 Among 4 Million PCR-Tested Individuals in Denmark in 2020: A Population-Level Observational Study. Lancet (2021) 397(10280):1204–12. doi: 10.1016/S0140-6736(21)00575-4 PMC7969130 33743221

[9] Hall VJ Foulkes S Charlett A Atti A Monk EJM Simmons R. SARS-CoV-2 Infection Rates of Antibody-Positive Compared With Antibody-Negative Health-Care Workers in England: A Large, Multicentre, Prospective Cohort Study (SIREN). Lancet (2021) 397(10283):1459–69. doi: 10.1016/S0140-6736(21)00675-9 PMC8040523 33844963

[10] Castro Dopico X Ols S Lore K Karlsson Hedestam GB. Immunity to SARS-CoV-2 Induced by Infection or Vaccination. J Intern Med (2021) 291(1):32–50. doi: 10.1111/joim.13372 34352148 PMC8447342

[11] Qureshi AI B WI Huang W Lobanova I Naqvi SH Shyu C-R. Reinfection With Severe Acute Respiratory Syndrome Coronavirus 2 (SARS-CoV-2) in Patients Undergoing Serial Laboratory Testing. Clin Infect Dis (2021) 74(2):294–300. doi: 10.1093/cid/ciab345 PMC8135382 33895814

[12] Zucman N Uhel F Descamps D Roux D Ricard JD. Severe Reinfection With South African SARS-CoV-2 Variant 501Y.V2: A Case Report. Clin Infect Dis (2021) 73(10):1945–46. doi: 10.1093/cid/ciab129 PMC7929064 33566076

[13] Resende PC Bezerra JF Teixeira Vasconcelos RH Arantes I Appolinario L Mendonca AC. Severe Acute Respiratory Syndrome Coronavirus 2 P.2 Lineage Associated With Reinfection Case, Brazil, June-October 2020. Emerg Infect Dis (2021) 27(7):1789–94. doi: 10.3201/eid2707.210401 PMC8237896 33883059

[14] Cavanaugh AM Spicer KB Thoroughman D Glick C Winter K. Reduced Risk of Reinfection With SARS-CoV-2 After COVID-19 Vaccination - Kentucky, May-June 2021. MMWR Morb Mortal Wkly Rep (2021) 70(32):1081–3. doi: 10.15585/mmwr.mm7032e1 PMC8360277 34383732

[15] Dan JM Mateus J Kato Y Hastie KM Yu ED Faliti CE. Immunological Memory to SARS-CoV-2 Assessed for Up to 8 Months After Infection. Science (2021) 371(6529):eabf4063. doi: 10.1126/science.abf4063 PMC7919858 33408181

[16] Kang CK Kim M Lee S Kim G Choe PG Park WB. Longitudinal Analysis of Human Memory T-Cell Response According to the Severity of Illness Up to 8 Months After Severe Acute Respiratory Syndrome Coronavirus 2 Infection. J Infect Dis (2021) 224(1):39–48. doi: 10.1093/infdis/jiab159 33755725 PMC8083680

[17] Xiang T Liang B Fang Y Lu S Li S Wang H. Declining Levels of Neutralizing Antibodies Against SARS-CoV-2 in Convalescent COVID-19 Patients One Year Post Symptom Onset. Front Immunol (2021) 12:708523:708523. doi: 10.3389/fimmu.2021.708523 34220870 PMC8242354

[18] Feng C Shi J Fan Q Wang Y Huang H Chen F. Protective Humoral and Cellular Immune Responses to SARS-CoV-2 Persist Up to 1 Year After Recovery. Nat Commun (2021) 12(1):4984. doi: 10.1038/s41467-021-25312-0 34404803 PMC8370972

[19] Kang CK Kim HR Song KH Keam B Choi SJ Choe PG. Cell-Mediated Immunogenicity of Influenza Vaccination in Patients With Cancer Receiving Immune Checkpoint Inhibitors. J Infect Dis (2020) 222(11):1902–9. doi: 10.1093/infdis/jiaa291 32479600

[20] Choe PG Kang CK Suh HJ Jung J Kang E Lee SY. Antibody Responses to SARS-CoV-2 at 8 Weeks Postinfection in Asymptomatic Patients. Emerg Infect Dis (2020) 26(10):2484–7. doi: 10.3201/eid2610.202211 PMC7510710 32579877

[21] Kim SW Kim SM Kim YK Kim JY Lee YM Kim BO. Clinical Characteristics and Outcomes of COVID-19 Cohort Patients in Daegu Metropolitan City Outbreak in 2020. J Korean Med Sci (2021) 36(1):e12. doi: 10.3346/jkms.2021.36.e12 33398946 PMC7781854

[22] Wu Z McGoogan JM. Characteristics of and Important Lessons From the Coronavirus Disease 2019 (COVID-19) Outbreak in China: Summary of a Report of 72314 Cases From the Chinese Center for Disease Control and Prevention. JAMA (2020) 323(13):1239–42. doi: 10.1001/jama.2020.2648 32091533

[23] Kang CK Kim M Lee S Kim G Choe PG Park WB. Longitudinal Analysis of Human Memory T-Cell Response According to the Severity of Illness Up to 8 Months After SARS-CoV-2 Infection. J Infect Dis (2021) 224(1):39–48. doi: 10.1093/infdis/jiab159 33755725 PMC8083680

[24] Wrapp D Wang N Corbett KS Goldsmith JA Hsieh CL Abiona O. Cryo-EM Structure of the 2019-Ncov Spike in the Prefusion Conformation. Science (2020) 367(6483):1260–3. doi: 10.1126/science.abb2507 PMC7164637 32075877

[25] Lee CH Romain G Yan W Watanabe M Charab W Todorova B. IgG Fc Domains That Bind C1q But Not Effector Fcgamma Receptors Delineate the Importance of Complement-Mediated Effector Functions. Nat Immunol (2017) 18(8):889–98. doi: 10.1038/ni.3770 PMC6015732 28604720

[26] Kang TH Lee CH Delidakis G Jung J Richard-Le Goff O Lee J. An Engineered Human Fc Variant With Exquisite Selectivity for FcgammaRIIIaV158 Reveals That Ligation of FcgammaRIIIa Mediates Potent Antibody Dependent Cellular Phagocytosis With GM-CSF-Differentiated Macrophages. Front Immunol (2019) 10:562. doi: 10.3389/fimmu.2019.00562 30984171 PMC6448688

[27] Kim HR Hong MS Dan JM Kang I. Altered IL-7Ralpha Expression With Aging and the Potential Implications of IL-7 Therapy on CD8+ T-Cell Immune Responses. Blood (2006) 107(7):2855–62. doi: 10.1182/blood-2005-09-3560 PMC1440715 16357322

[28] Grifoni A Weiskopf D Ramirez SI Mateus J Dan JM Moderbacher CR. Targets of T Cell Responses to SARS-CoV-2 Coronavirus in Humans With COVID-19 Disease and Unexposed Individuals. Cell (2020) 181(7):1489–501.e15. doi: 10.1016/j.cell.2020.05.015 32473127 PMC7237901

[29] Laidlaw BJ Ellebedy AH. The Germinal Centre B Cell Response to SARS-CoV-2. Nat Rev Immunol (2022) 22(1):7–18. doi: 10.1038/s41577-021-00657-1 34873279 PMC8647067

[30] Khoury DS Cromer D Reynaldi A Schlub TE Wheatley AK Juno JA. Neutralizing Antibody Levels are Highly Predictive of Immune Protection From Symptomatic SARS-CoV-2 Infection. Nat Med (2021) 27(7):1205–11. doi: 10.1038/s41591-021-01377-8 34002089

[31] Legros V Denolly S Vogrig M Boson B Siret E Rigaill J. A Longitudinal Study of SARS-CoV-2-Infected Patients Reveals a High Correlation Between Neutralizing Antibodies and COVID-19 Severity. Cell Mol Immunol (2021) 18(2):318–27. doi: 10.1038/s41423-020-00588-2 PMC7786875 33408342

[32] Chen W Zhang J Qin X Wang W Xu M Wang LF. SARS-CoV-2 Neutralizing Antibody Levels are Correlated With Severity of COVID-19 Pneumonia. BioMed Pharmacother (2020) 130:110629. doi: 10.1016/j.biopha.2020.110629 33406577 PMC7425713

[33] Butler AL Fallon JK. Alter G. A Sample-Sparing Multiplexed ADCP Assay. Front Immunol (2019) 10:1851:1851. doi: 10.3389/fimmu.2019.01851 31456799 PMC6700248

[34] Tay MZ Wiehe K Pollara J. Antibody-Dependent Cellular Phagocytosis in Antiviral Immune Responses. Front Immunol (2019) 10:332. doi: 10.3389/fimmu.2019.00332 30873178 PMC6404786

[35] Choe PG Kang CK Kim KH Yi J Kim ES Park SW. Persistence of Neutralizing Antibody Response Up to One Year After Asymptomatic or Symptomatic SARS-CoV-2 Infection. J Infect Dis (2021) 224(6):1097–99. doi: 10.1093/infdis/jiab339 34166506

[36] Sun B Feng Y Mo X Zheng P Wang Q Li P. Kinetics of SARS-CoV-2 Specific IgM and IgG Responses in COVID-19 Patients. Emerg Microbes Infect (2020) 9(1):940–8. doi: 10.1080/22221751.2020.1762515 PMC7273175 32357808

[37] Liu Y Yan LM Wan L Xiang TX Le A Liu JM. Viral Dynamics in Mild and Severe Cases of COVID-19. Lancet Infect Dis (2020) 20(6):656–7. doi: 10.1016/S1473-3099(20)30232-2 PMC7158902 32199493

[38] Kang CK Han GC Kim M Kim G Shin HM Song KH. Aberrant Hyperactivation of Cytotoxic T-Cell as a Potential Determinant of COVID-19 Severity. Int J Infect Dis (2020) 97:313–21. doi: 10.1016/j.ijid.2020.05.106 PMC7261468 32492530

[39] Zohar T Alter G. Dissecting Antibody-Mediated Protection Against SARS-CoV-2. Nat Rev Immunol (2020) 20(7):392–4. doi: 10.1038/s41577-020-0359-5 PMC7278217 32514035

[40] Li D Sempowski GD Saunders KO Acharya P Haynes BF. SARS-CoV-2 Neutralizing Antibodies for COVID-19 Prevention and Treatment. Annu Rev Med (2022) 73:1–16. doi: 10.1146/annurev-med-042420-113838 34428080

[41] Yamin R Jones AT Hoffmann HH Schafer A Kao KS Francis RL. Fc-Engineered Antibody Therapeutics With Improved Anti-SARS-CoV-2 Efficacy. Nature (2021) 599(7885):465–70. doi: 10.1038/s41586-021-04017-w PMC9038156 34547765

[42] Ma L Sahu SK Cano M Kuppuswamy V Bajwa J McPhatter J. Increased Complement Activation is a Distinctive Feature of Severe SARS-CoV-2 Infection. bioRxiv (2021) 6(59):eabh2259. doi: 10.1101/2021.02.22.432177 PMC8158979 34446527

[43] Holter JC Pischke SE de Boer E Lind A Jenum S Holten AR. Systemic Complement Activation is Associated With Respiratory Failure in COVID-19 Hospitalized Patients. Proc Natl Acad Sci U S A (2020) 117(40):25018–25. doi: 10.1073/pnas.2010540117 PMC7547220 32943538

[44] Skendros P Mitsios A Chrysanthopoulou A Mastellos DC Metallidis S Rafailidis P. Complement and Tissue Factor-Enriched Neutrophil Extracellular Traps are Key Drivers in COVID-19 Immunothrombosis. J Clin Invest (2020) 130(11):6151–7. doi: 10.1172/JCI141374 PMC7598040 32759504

[45] Li Q Chen Z. An Update: The Emerging Evidence of Complement Involvement in COVID-19. Med Microbiol Immunol (2021) 210(2-3):101–9. doi: 10.1007/s00430-021-00704-7 PMC8019074 33811541

[46] Kang S Yang M He S Wang Y Chen X Chen YQ. A SARS-CoV-2 Antibody Curbs Viral Nucleocapsid Protein-Induced Complement Hyperactivation. Nat Commun (2021) 12(1):2697. doi: 10.1038/s41467-021-23036-9 33976229 PMC8113585

[47] Sterlin D Mathian A Miyara M Mohr A Anna F Claer L. IgA Dominates the Early Neutralizing Antibody Response to SARS-CoV-2. Sci Transl Med (2021) 13(577):eabd2223. doi: 10.1126/scitranslmed.abd2223 PMC7857408 33288662

[48] Huber VC Lynch JM Bucher DJ Le J Metzger DW. Fc Receptor-Mediated Phagocytosis Makes a Significant Contribution to Clearance of Influenza Virus Infections. J Immunol (2001) 166(12):7381–8. doi: 10.4049/jimmunol.166.12.7381 11390489

[49] Kaneko N Kuo HH Boucau J Farmer JR Allard-Chamard H Mahajan VS. Loss of Bcl-6-Expressing T Follicular Helper Cells and Germinal Centers in COVID-19. Cell (2020) 183(1):143–57 e13. doi: 10.1016/j.cell.2020.08.025 32877699 PMC7437499

[50] Centers for Disease Control and Prevention. Vaccines for COVID-19 . Available at: https://www.cdc.gov/coronavirus/2019-ncov/vaccines/index.html. 34009769

[51] Anichini G Terrosi C Gandolfo C Gori Savellini G Fabrizi S Miceli GB. SARS-CoV-2 Antibody Response in Persons With Past Natural Infection. N Engl J Med (2021) 385(1):90–2. doi: 10.1056/NEJMc2103825 PMC8063888 33852796

[52] Ebinger JE Fert-Bober J Printsev I Wu M Sun N Prostko JC. Antibody Responses to the BNT162b2 mRNA Vaccine in Individuals Previously Infected With SARS-CoV-2. Nat Med (2021) 27(6):981–4. doi: 10.1038/s41591-021-01325-6 PMC8205849 33795870

