# Corrigendum: Distinct Immune Response at 1 Year Post-COVID-19 According to Disease Severity

**DOI:** 10.3389/fimmu.2022.929770

**Published:** 2022-05-24

**Authors:** Chang Kyung Kang, Minji Kim, Jisu Hong, Gwanghun Kim, Soojin Lee, Euijin Chang, Pyoeng Gyun Choe, Nam Joong Kim, Ik Soo Kim, Jun-Young Seo, Daesub Song, Dong-Sup Lee, Hyun Mu Shin, Yong-Woo Kim, Chang-Han Lee, Wan Beom Park, Hang-Rae Kim, Myoung-don Oh

**Affiliations:** ^1^ Department of Internal Medicine, Seoul National University College of Medicine, Seoul, South Korea; ^2^ Department of Biomedical Sciences, Seoul National University College of Medicine, Seoul, South Korea; ^3^ Department of Anatomy & Cell Biology, Seoul National University College of Medicine, Seoul, South Korea; ^4^ BrainKorea21 (BK21) FOUR Biomedical Science Project, Seoul National University College of Medicine, Seoul, South Korea; ^5^ Department of Pharmacology, Seoul National University College of Medicine, Seoul, South Korea; ^6^ Department of Microbiology, School of Medicine, Gachon University, Incheon, South Korea; ^7^ Severance Biomedical Science Institute, Yonsei University College of Medicine, Seoul, South Korea; ^8^ BrainKorea21 (BK21) Project for Medical Science, Yonsei University College of Medicine, Seoul, South Korea; ^9^ College of Pharmacy, Korea University, Sejong, South Korea; ^10^ Medical Research Institute, Seoul National University College of Medicine, Seoul, South Korea; ^11^ Wide River Institute of Immunology, Seoul National University, Hongcheon, South Korea

**Keywords:** SARS-CoV-2, antibody, phagocytosis, memory B cells, memory T cells

In the original article, there was a mistake in **Figure 3** as published. **Figure 3D** was repeatedly used instead of **Figure 3F** while reformatting the **Figure 3** plots during the revision process. The corrected **Figure 3** appears below.

**Figure 3:**
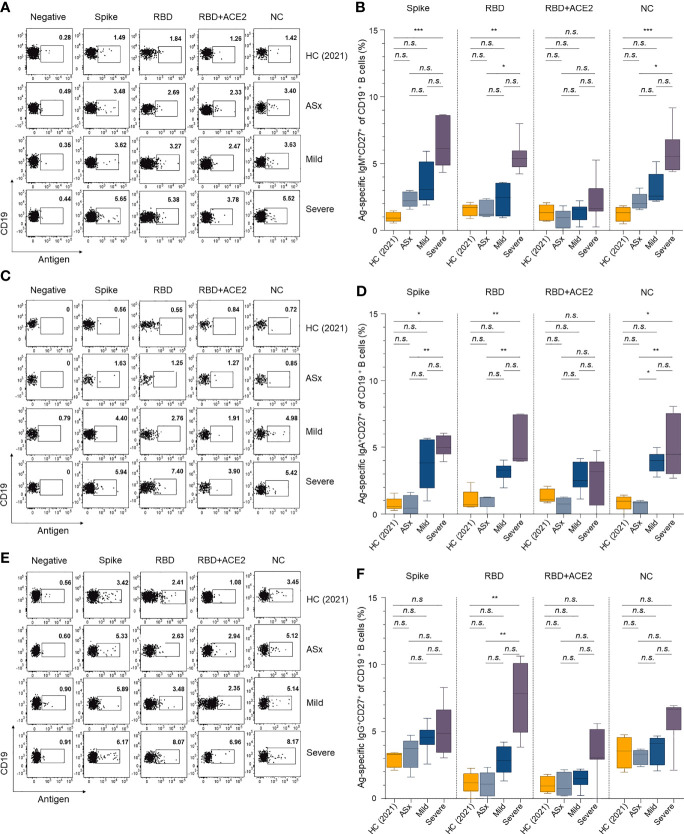
Memory B-cell responses against SARS-CoV-2 according to the severity of illness. **(A, C, E)** Representative gating strategy for IgM+, IgA+, and IgG+ antigen-specific memory (CD27+ CD19+) B cells. **(B, D, F)** Frequencies of IgM+, IgA+, and IgG+ antigen-specific memory B cells according to the severity of illness and different antigens. Statistical analysis was performed using the Kruskal–Wallis rank-sum test with Dunn’s post hoc test in GraphPad Prism (n.s.: P > 0.05, *P < 0.05, **P < 0.01, ***P < 0.001).

The authors apologize for this error and state that this does not change the scientific conclusions of the article in any way. The original article has been updated.

## Publisher’s Note

All claims expressed in this article are solely those of the authors and do not necessarily represent those of their affiliated organizations, or those of the publisher, the editors and the reviewers. Any product that may be evaluated in this article, or claim that may be made by its manufacturer, is not guaranteed or endorsed by the publisher.

