# Supplementary material for: Distinct Immune Response at 1 Year Post-COVID-19 According to Disease Severity
Source: Front Immunol. 2022 Mar 21;13:830433. doi: 10.3389/fimmu.2022.830433 (PMC8980227; doi:10.3389/fimmu.2022.830433)

Supplementary Figure S1

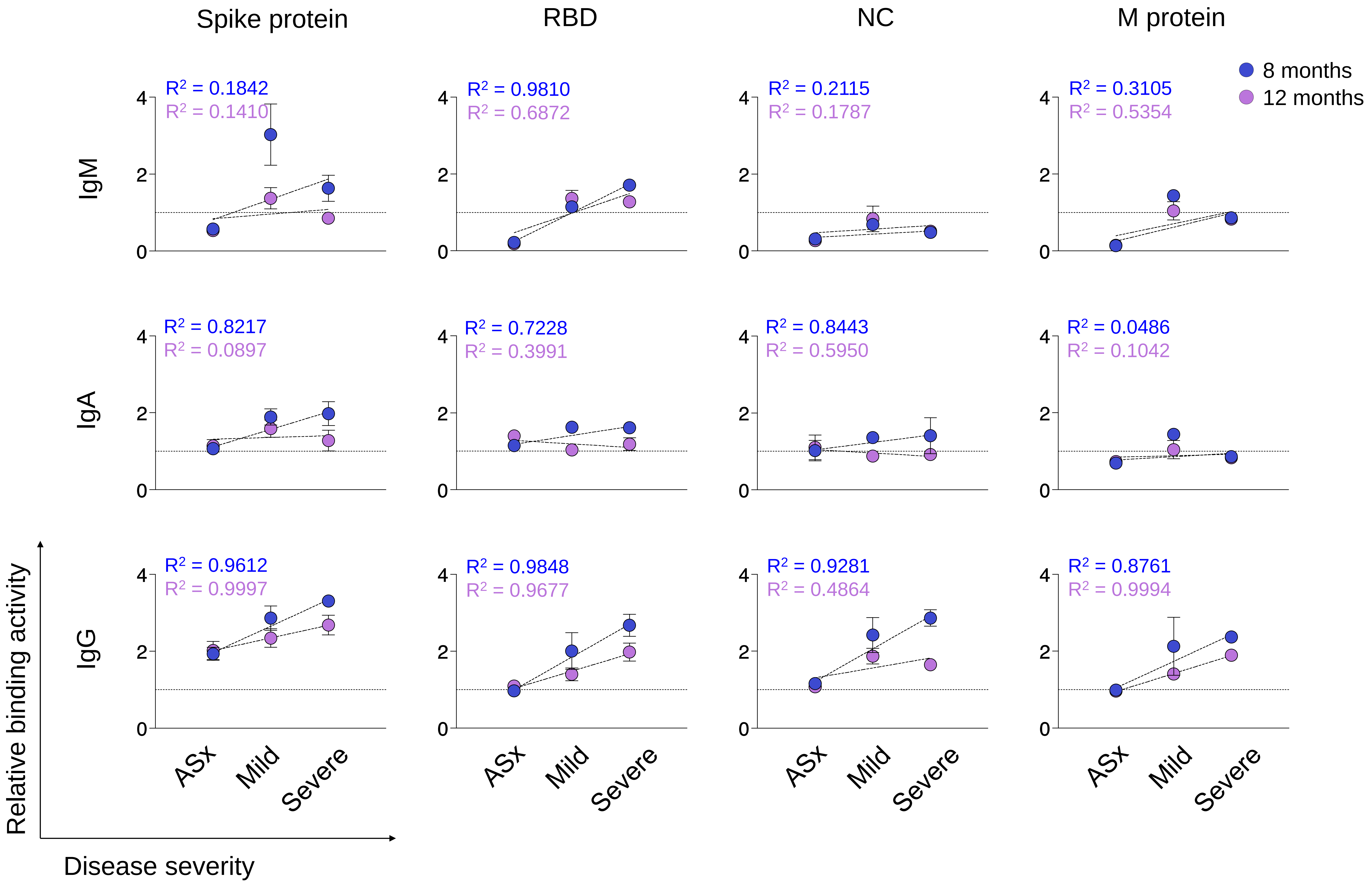

Supplementary Figure S2

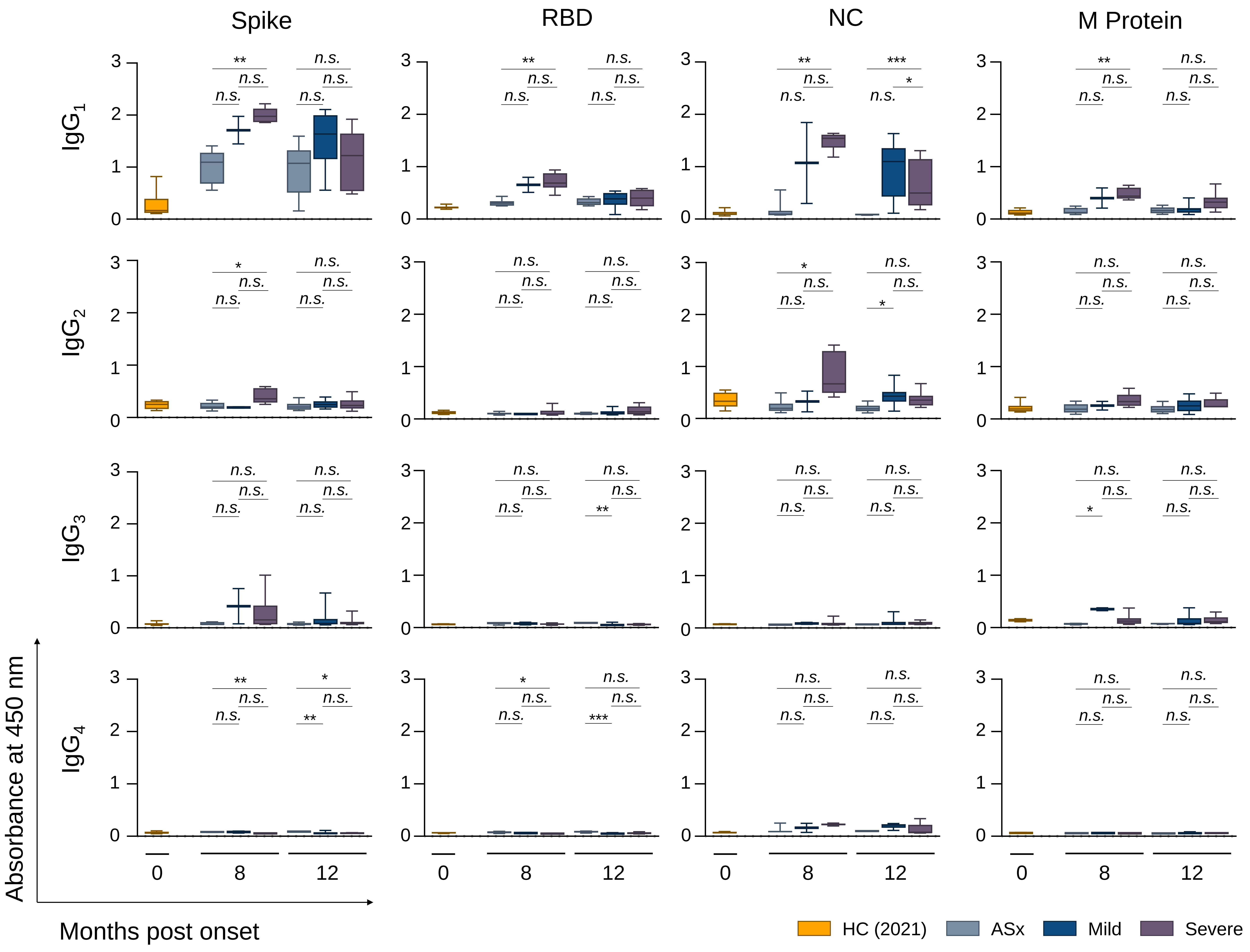

Supplementary Figure S3

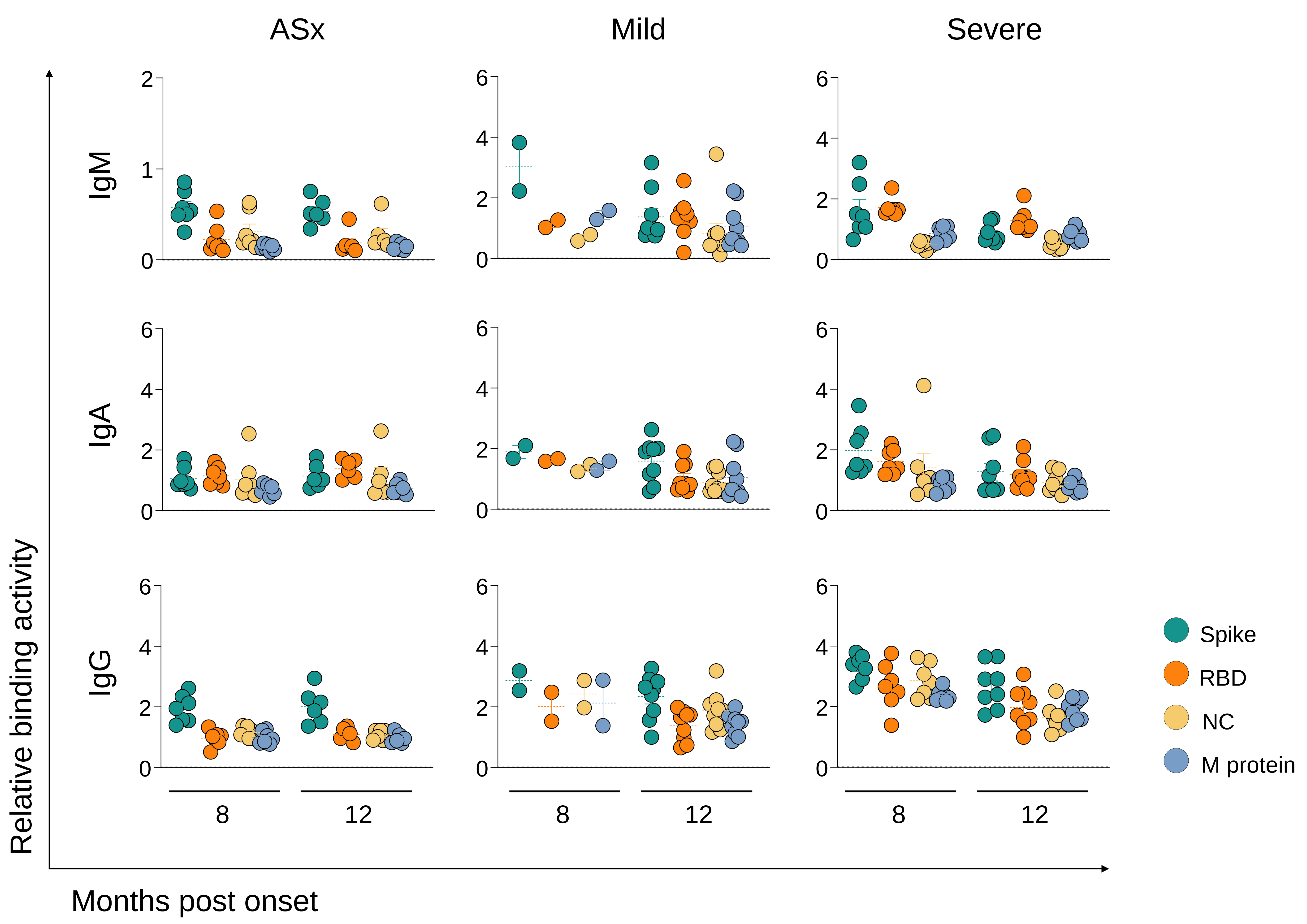

Supplementary Figure S4

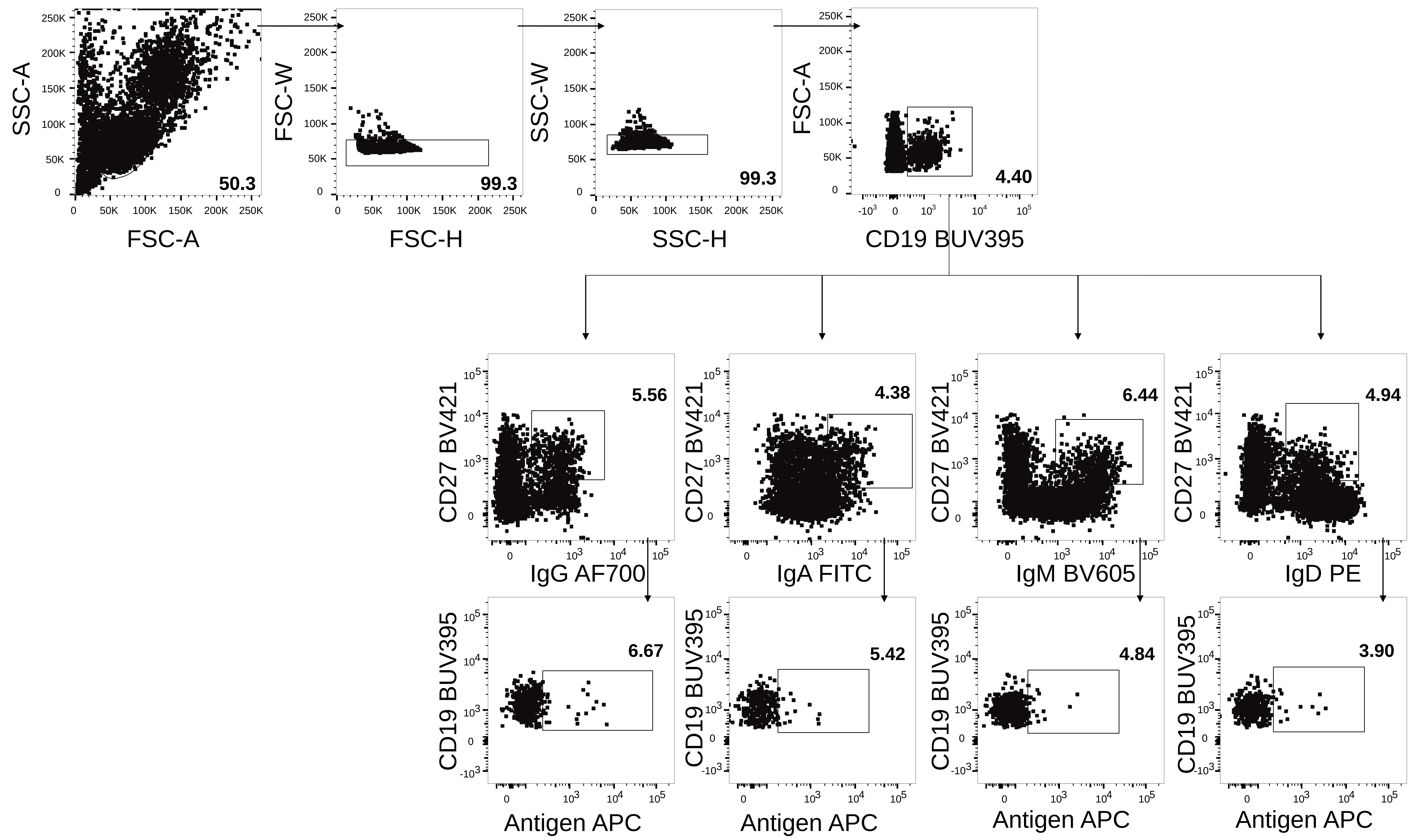

Supplementary Figure S5

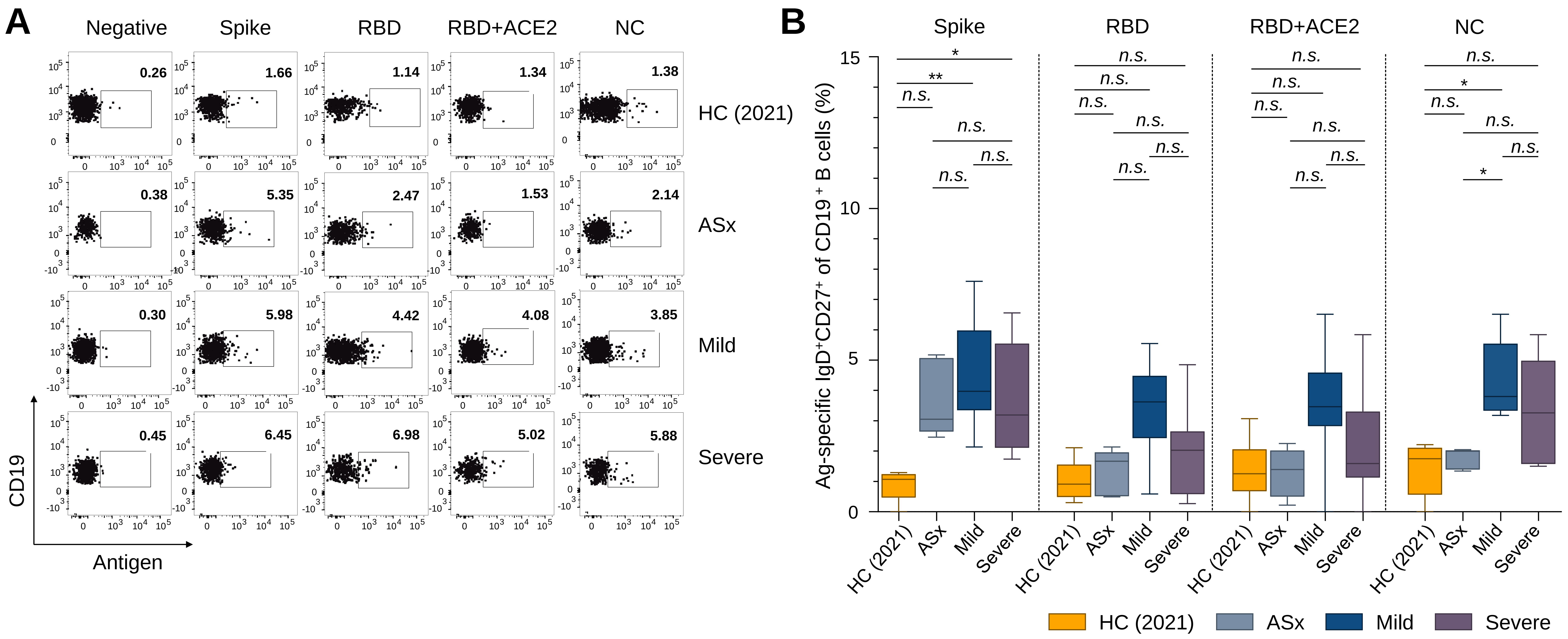

Supplementary Figure S6

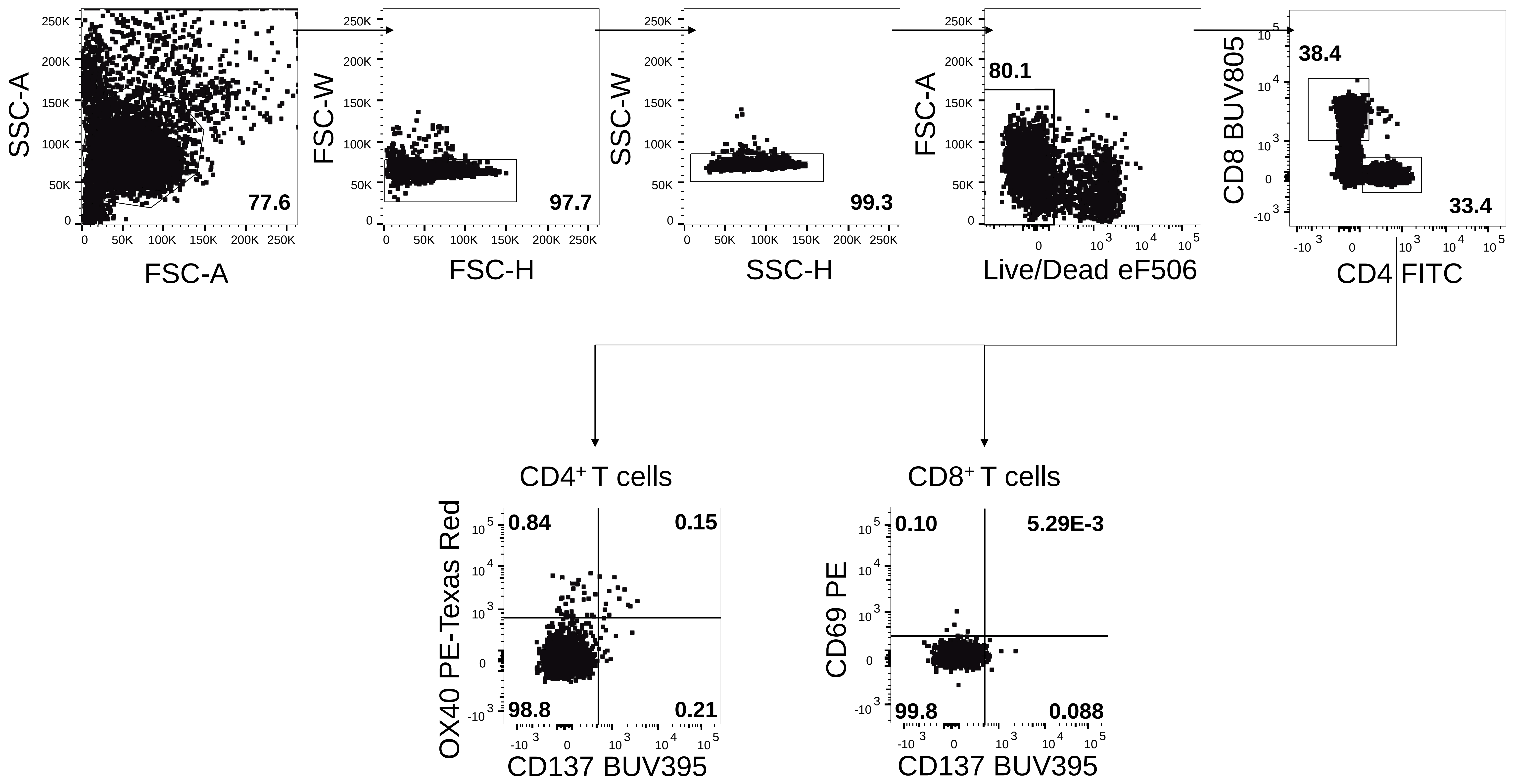

Supplement: Supplementary Figure S1 — Correlation of antibody response and severity of COVID-19 patients. Each antibody’s relative binding activity to SARS-CoV-2-specific protein was presented with severity of COVID-19 patients. Coefficient of determination (R2) was calculated by linear regression analysis. ASx: asymptomatic (8-month: n = 7, 12-month: n = 6), Mild (8-month: n = 2, 12-month: n = 9), Severe (8-month: n = 7, 12-month: n = 8). [file DataSheet_1.pdf]
