# Supplementary material for: Distinct Immune Response at 1 Year Post-COVID-19 According to Disease Severity
Source: Front Immunol. 2022 Mar 21;13:830433. doi: 10.3389/fimmu.2022.830433 (PMC8980227; doi:10.3389/fimmu.2022.830433)
Supplement: Supplementary file 2 [file Table_1.docx]

**Supplementary Table S1** Clinical characteristics of each patient in this study

| Number | Severity | Age | Gender | DM | HTN | Maximal  O_2_ demand | Treatment | Days of sample collection from the onset of COVID-19 | |
| --- | --- | --- | --- | --- | --- | --- | --- | --- | --- |
|  |  |  |  |  |  |  |  | 8 months | 12 months |
| A1 | Asymptomatic | 23 | M | - | - | - | - | 229^a,b^ | - |
| A2 | Asymptomatic | 25 | M | - | - | - | - | 234^a,b^ | 350 |
| A3 | Asymptomatic | 28 | M | - | - | - | - | 231^a,b^ | 343^a^ |
| A4 | Asymptomatic | 26 | F | - | - | - | - | 235^a,b^ | 351 |
| A5 | Asymptomatic | 21 | F | - | - | - | - | 230^a,b^ | 351^a,b^ |
| A6 | Asymptomatic | 20 | M | - | - | - | - | 231^a,b^ | 354 |
| A7 | Asymptomatic | 26 | M | - | - | - | - | 230^a,b^ | 355 |
| M1 | Mild | 55 | M | + | - | - | - | - | 382^a,b^ |
| M2 | Mild | 53 | F | - | - | - | - | - | 378 |
| M3 | Mild | 24 | M | - | - | - | - | - | 378^a,b^ |
| M4 | Mild | 72 | F | - | - | - | Remdesivir, baricitinib | 192^a,b^ | 315^b^ |
| M5 | Mild | 41 | F | - | - | - | - | 232^a,b^ | 352 |
| M6 | Mild | 34 | F | - | - | - | - | - | 326 |
| M7 | Mild | 69 | F | - | + | - |  | - | 370 |
| M8 | Mild | 43 | M | + | + | - |  | - | 347 |
| M9 | Mild | 60 | F | + | - | - | - | - | 383 |
| S1 | Severe | 39 | M | - | - | NP | - | - | 323 |
| S2 | Severe | 67 | M | + | + | NP | Remdesivir, baricitinib | 195^a,b^ | 358 |
| S3 | Severe | 48 | M | - | - | NP | Remdesivir, steroid | 220^a,b^ | 318 |
| S4 | Severe | 69 | F | - | + | HFNC | Remdesivir | 197^a,b^ | 320^a,b^ |
| S5 | Severe | 51 | M | - | + | HFNC | Remdesivir | 197^a,b^ | 367 |
| S6 | Severe | 65 | M | + | - | HFNC | Remdesivir, baricitinib | 188^a,b^ | 358 |
| S7 | Severe | 76 | F | - | + | MV | Remdesivir | 185^a,b^ | 353 |
| S8 | Severe | 61 | M | + | + | MV | Remdesivir, baricitinib, steroid | 198^a,b^ | 368 |

*DM, diabetes mellitus; HTN, hypertension; HFNC, high flow nasal canula; NP, nasal prong; MV, mechanical ventilation*

^a^ Could not be analyzed for memory T-cell responses

^b^ Could not be analyzed for memory B-cell responses
